# Functionalized silk spheres selectively and effectively deliver a cytotoxic drug to targeted cancer cells in vivo

**DOI:** 10.1186/s12951-020-00734-y

**Published:** 2020-12-01

**Authors:** Anna Florczak, Tomasz Deptuch, Anna Lewandowska, Karolina Penderecka, Elzbieta Kramer, Andrzej Marszalek, Andrzej Mackiewicz, Hanna Dams-Kozlowska

**Affiliations:** 1 Chair of Medical Biotechnology, Poznan University of Medical Sciences, 15 Garbary St, 61-866 Poznan, Poland; 2 Department of Diagnostics and Cancer Immunology, Greater Poland Cancer Centre, 15 Garbary St, 61-866 Poznan, Poland; 3 Department of Tumor Pathology, Greater Poland Cancer Centre, 15 Garbary St, 61-866 Poznan, Poland; 4 Department of Tumor Pathology and Prophylaxis, Poznan University of Medical Sciences, 15 Garbary St, 61-866 Poznan, Poland

**Keywords:** Silk, Particles, Functionalization, Bioengineering, Targeted drug delivery, Cancer, Mouse cancer models

## Abstract

**Background:**

Chemotherapy is often a first-line therapeutic approach for the treatment of a wide variety of cancers. Targeted drug delivery systems (DDSs) can potentially resolve the problem of chemotherapeutic drug off-targeting effects. Herein, we examined in vivo models to determine the efficacy of Her2-targeting silk spheres (H2.1MS1) as DDSs for delivering doxorubicin (Dox) to Her2-positive and Her2-negative primary and metastatic mouse breast cancers.

**Results:**

The specific accumulation of H2.1MS1 spheres was demonstrated at the site of Her2-positive cancer. Dox delivered only by functionalized H2.1MS1 particles selectively inhibited Her2-positive cancer growth in primary and metastatic models. Moreover, the significant effect of the Dox dose and the frequency of treatment administration on the therapeutic efficacy was indicated. Although the control MS1 spheres accumulated in the lungs in Her2-positive metastatic breast cancer, the Dox-loaded MS1 particles did not treat cancer. Histopathological examination revealed no systemic toxicity after multiple administrations and at increased doses of Dox-loaded silk spheres. Although the studies were performed in immunocompetent mice, the H2.1MS1 silk spheres efficiently delivered the drug, which exerted a therapeutic effect.

**Conclusion:**

Our results indicated that functionalized silk spheres that enable cell-specific recognition, cellular internalization, and drug release represent an efficient strategy for cancer treatment in vivo.
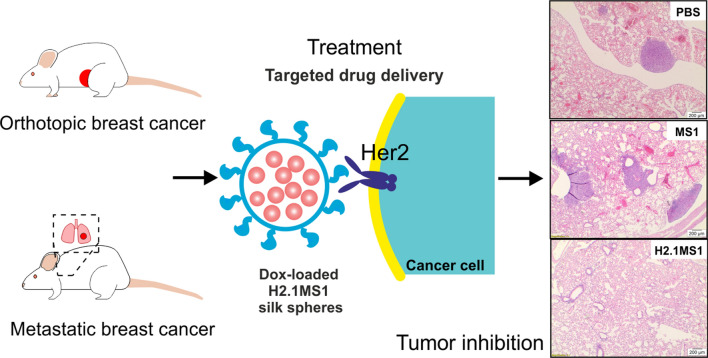

## Background

Chemotherapy is often a first-line therapeutic approach for the treatment of a wide variety of cancers [1, 2]. However, nonspecific delivery of chemotherapeutic agents leads to undesirable side effects in healthy tissues and dosages that are insufficient to kill cancer cells. Therefore, various targeted drug delivery systems have been investigated [3] to overcome these difficulties.

Nanomaterial-based drug delivery systems (DDSs) deliver therapeutic agents to specific organs or tissues using nanoscale particles to circumvent the nonspecific biodistribution of free drugs [4, 5]. A DDS should be characterized by simple preparation, high drug loading capacity, and excellent stability [6, 7]. Silk biomaterials are considered to be great candidates for various biomedical applications, as they are biocompatible, biodegradable, nontoxic, and straightforward to prepare. Silks can self-assemble to assume several morphologies, such as films, scaffolds, hydrogels, microcapsules, and spheres [8]. Moreover, genetic engineering enables the functionalization of the bioengineered silk by adding sequences encoding peptides, domains, or proteins that confer a function and expand the biomaterial applications, e.g., targeting of tumor cells to increase therapeutic efficacy [9, 10]. Spheres made of functionalized silk are extremely promising as carriers of anticancer agents [8, 11].

Extensive studies have shown that nanoparticles can enter tumor sites through both passive and active targeting processes. Passive targeting takes advantage of the enhanced permeability and retention (EPR) effect for preferential tumor accumulation of nanoparticles [6, 12]. However, some unresolved problems impede the therapeutic effect of the passively targeted DDS that mainly involve the insufficient accumulation of nanomedicines at targeted sites [13, 14]. The active targeting of tumors can be achieved by utilizing specific ligand–receptor interactions in the drug or drug carrier design. Active targeting provides a promising way for drug nanocarriers to target neoplasms.

One of the proposed mechanisms of tumorigenesis and the acquisition of resistance to cancer treatment concerns a family of four human plasma membrane receptors, the ErbB tyrosine kinase receptor family (Her1-4) [15]. In particular, Her2 is present in numerous cancers and significantly affects the effectiveness of treatment [16, 17]. Her2 regulates the cellular proliferation and survival of cells by dimerizing with other ErbB receptors, which results in the autophosphorylation of its tyrosine residues and initiation of a variety of signaling pathways [17, 18]. The amplification of the HER2/*neu* gene results in Her2 overexpression, which occurs in approximately 20%-30% of ovarian and breast cancers and is associated with tumorigenesis, metastasis and a poor prognosis [18, 19]. The humanized recombinant monoclonal antibody trastuzumab (Herceptin™) was the first anti-Her2 agent approved by the US Food and Drug Administration in 1998 for the treatment of Her2-positive breast tumors [20, 21]. However, at least 50% of Her2-positive tumors develop resistance to trastuzumab [22], and trastuzumab-targeted therapy is considered to be potentially cardiotoxic [23]. Therefore, since the introduction of trastuzumab to the market, many anti-Her2 antibodies and small molecular inhibitors affecting the Her2 signaling pathway have been evaluated in clinical trials [24, 25]. Moreover, development has occurred in the design of targeted DDS with immobilized Her2 ligands, including Fab antibody fragments [26, 27], scFv (single-chain variable) fragments [28, 29], affibody molecules [30, 31] and peptides [32–34].

Doxorubicin (Dox) is a routinely used and effective chemotherapeutic anthracycline drug for Her2-positive cancer therapy [35]. Nevertheless, clinical applications of Dox have been limited by the poor water solubility and dose-limiting off-target side effects of Dox [36], which are due mainly to its cardiotoxicity [37]. Moreover, many cancers exhibit either intrinsic or acquired resistance to Dox treatment due to the increased expression of drug efflux-associated proteins [38, 39]. Thus, combinational drug regimens may overcome drug resistance.

We designed a DDS based on bioengineered silk spheres functionalized with a Her2-binding peptide (H2.1) named H2.1MS1 [40]. The H2.1MS1 and control MS1 nanoparticles were carefully characterized in terms of size, morphology, structure, zeta potential, stability, and drug loading/release capacity, cytotoxicity, and the ability to selectively deliver doxorubicin (Dox) to the target cells [40–43]. In vitro studies indicated that the H2.1 peptide enabled the endocytosis-mediated internalization of functionalized spheres into target cells upon binding with the Her2 receptor [40, 44]. The spheres were degraded enzymatically in lysosomes, and the encapsulated cytostatic drug was released from the silk nanoparticles. The drug localized to the nucleus, where it induced high cytotoxicity in target cells [40]. Drug delivery was highly selective, as the drug-loaded silk-based delivery system did not kill healthy cells [40]. Moreover, control MS1 spheres were significantly less effective at delivering the drug to cancer cells compared with the targeted drug delivery observed when using H2.1MS1 particles [40].

Herein, for the first time, we examined the in vivo efficacy of functionalized silk spheres as a drug delivery system for cancer treatment. Her2-positive and Her2-negative breast cancer cells were used to mimic both primary and metastatic breast cancer models in immunocompetent mice. The specific accumulation of H2.1MS1 spheres at the sites of Her2(+) tumors was demonstrated. Dox that was delivered only by functionalized H2.1MS1 spheres selectively inhibited Her2(+) tumor growth in both models. Moreover, these studies indicated the significant effect of the Dox dose and the frequency of treatment administration on the therapeutic efficacy. Histopathological examination revealed no systemic toxicity when Dox-loaded silk spheres were applied.

## Results

### Analysis of the Her2(+) tumor-targeting properties of the functionalized silk spheres in vitro

**Fig. 1:**
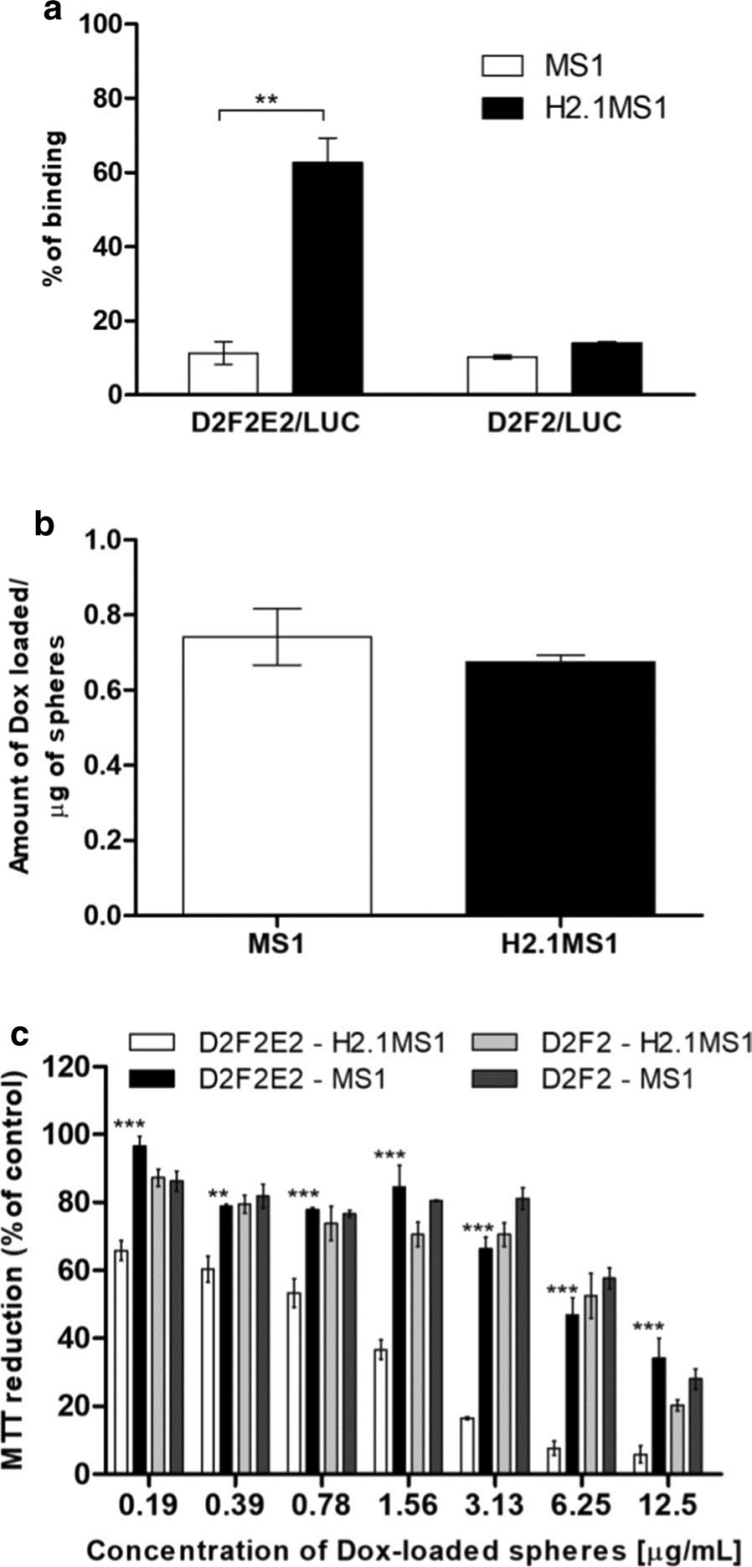
In vitro characterization of Her2 tumor-targeting properties of the functionalized silk particles. **a** Flow cytometry analysis of the binding of silk spheres to the cancer cells. Murine Her2-overexpressing cells (D2F2E2/LUC) and control Her2-negative cells (D2F2/LUC) were incubated with spheres made of functionalized (H2.1MS1) or control (MS1) silks conjugated with a fluorophore (ATTO647N) and then analyzed cytometrically. **b** Doxorubicin loading into silk particles. The amount of Dox incorporated into the MS1 and H2.1MS1 spheres are presented. **c** Cytotoxicity study by the MTT assay. Her2-overexpressing (D2F2E2/Luc) cells and control cells (D2F2/Luc) were cultured in the presence of the silk spheres loaded with Dox (MS1 or H2.1MS1). The percentage of the MTT reduction was calculated in reference to non-treated control cells. The results are expressed as the mean of three independent experiments ± SEM. (**) indicates statistical significance with p < 0.01 and (***) p < 0.001

To determine the tumor-targeting ability of H2.1MS1 spheres in vivo, Her2-overexpressing (Her2(+)) and Her2-negative (Her2(−)) mouse breast tumor cells (D2F2E2/LUC and D2F2/LUC cell lines, respectively) were used in this study. First, the D2F2E2/LUC and D2F2/LUC cells were prepared as indicated in the Additional files. Clones D2F2E2/LUC #1 and D2F2/LUC #5 displayed the highest levels of luminescence and proliferation rates similar to those of their native counterparts (Additional files 2 and 3: Table S1, Figure S1, respectively). The detailed examination of selected clones indicated that D2F2/LUC cells proliferated at a rate that was significantly faster than that of D2F2E2/LUC cells (Additional file 4: Figure S2). Moreover, we showed that there was sixfold higher cellular binding of functionalized H2.1MS1 spheres to D2F2E2 cancer cells than that of control MS1 spheres (Fig. 1a). There was approximately 10% nonspecific binding of H2.1MS1 and MS1 particles to Her2(−) D2F2 cells (Fig. 1a). The total amount of doxorubicin (Dox) incorporated into the control and functionalized spheres was similar for both types of particles (approximately 0.70 mg Dox/mg of spheres) (Fig. 1b). The Dox delivered by the H2.1MS1 spheres significantly reduced the viability of D2F2E2/LUC cells in vitro compared to the viability of control cells and when treated with control spheres (Fig. 1c).

### Biodistribution of functionalized spheres in a model of orthotopic breast cancer

A preliminary examination was performed to analyze the biodistribution of H2.1MS1 spheres in the Her2(+) and Her2(−) orthotopic breast cancer models in BALB/c mice. The tumor-bearing mice were injected intravenously with fluorescently labeled H2.1MS1 spheres on days 0, 3, and 6. As a control, a fluorescently labeled anti-Her2 antibody was applied (Herceptin).

**Fig. 2:**
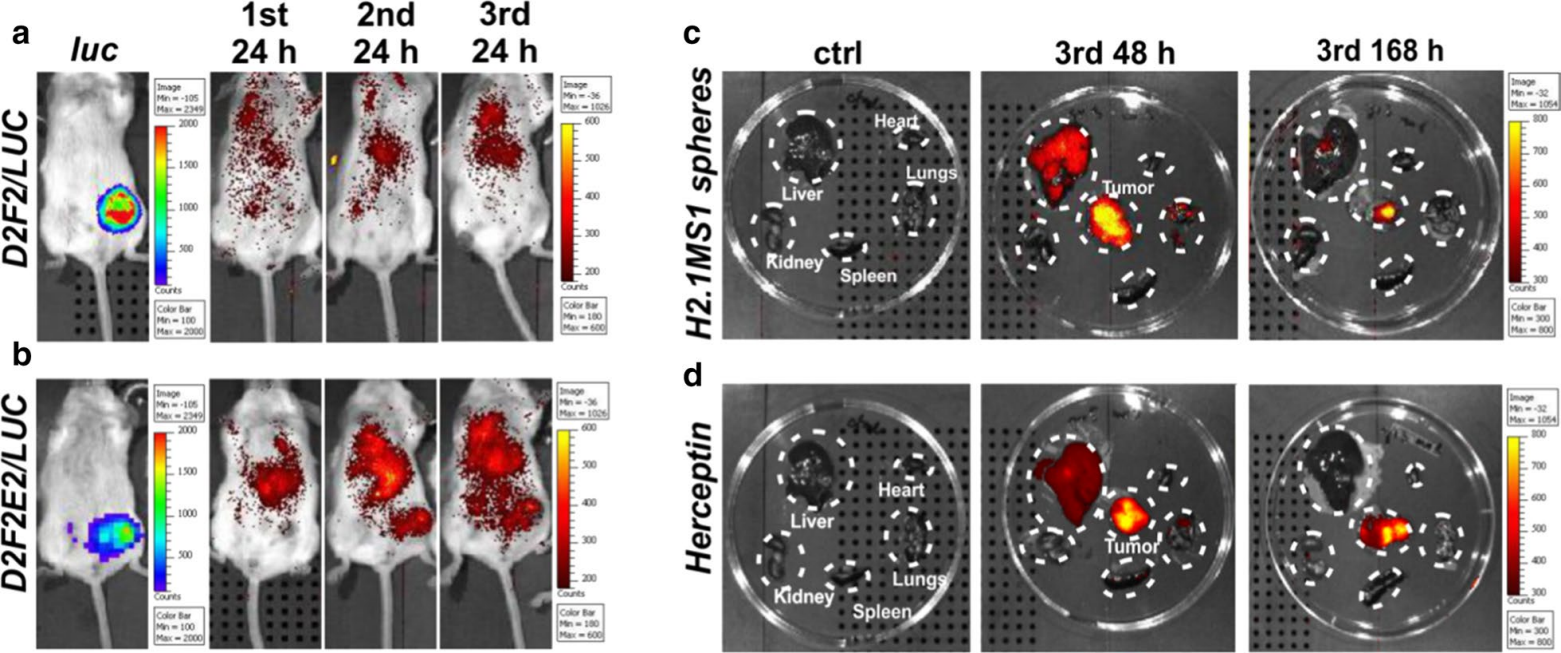
Representative images indicating the biodistribution of functionalized silk spheres in Her2(+) and Her2(−) mouse orthotopic breast cancer models. Biodistribution of H2.1MS1 spheres over time after intravenous injection of BALB/c mice, which developed **a** Her2(−) D2F2 tumors and **b** Her2(+) D2F2E2 tumors. Biodistribution of **c** H2.1MS1 spheres and **d** Herceptin 48 and 168 h after the 3rd intravenous injection in mice that developed D2F2E2 tumors. Organs and tumors were excised and imaged. Signal detection was performed by using the IVIS Spectrum system to estimate the luminescence intensity of the tumors and the fluorescence intensity of the ATTO647N-labeled H2.1MS1 spheres and Herceptin at wavelengths of 640/680 nm

There was no accumulation of functionalized spheres in Her2(−) tumors after intravenous administration (Fig. 2a). At 24 h after the second injection, a notable accumulation of H2.1MS1 particles was found in the Her2(+) tumors (Fig. 2b). To further determine the localization of functionalized silk spheres in mice, the organs were collected 48 and 168 h after the 3rd injection of the functionalized silk spheres and were then imaged using an IVIS imaging system. Initially, the H2.1MS1 particles, in addition to accumulation in tumors, were localized in the lungs and liver; however, in time, the fluorescent signal in organs disappeared yet was still observed in the tumor (Fig. 2c). Moreover, Herceptin accumulated in the D2F2E2 tumors, confirming the presence of the Her2 molecule in the applied mouse model of breast cancer (Fig. 2d).

### Antitumor efficacy of Dox-loaded functionalized silk spheres in a model of orthotopic breast cancer

**Fig. 3:**
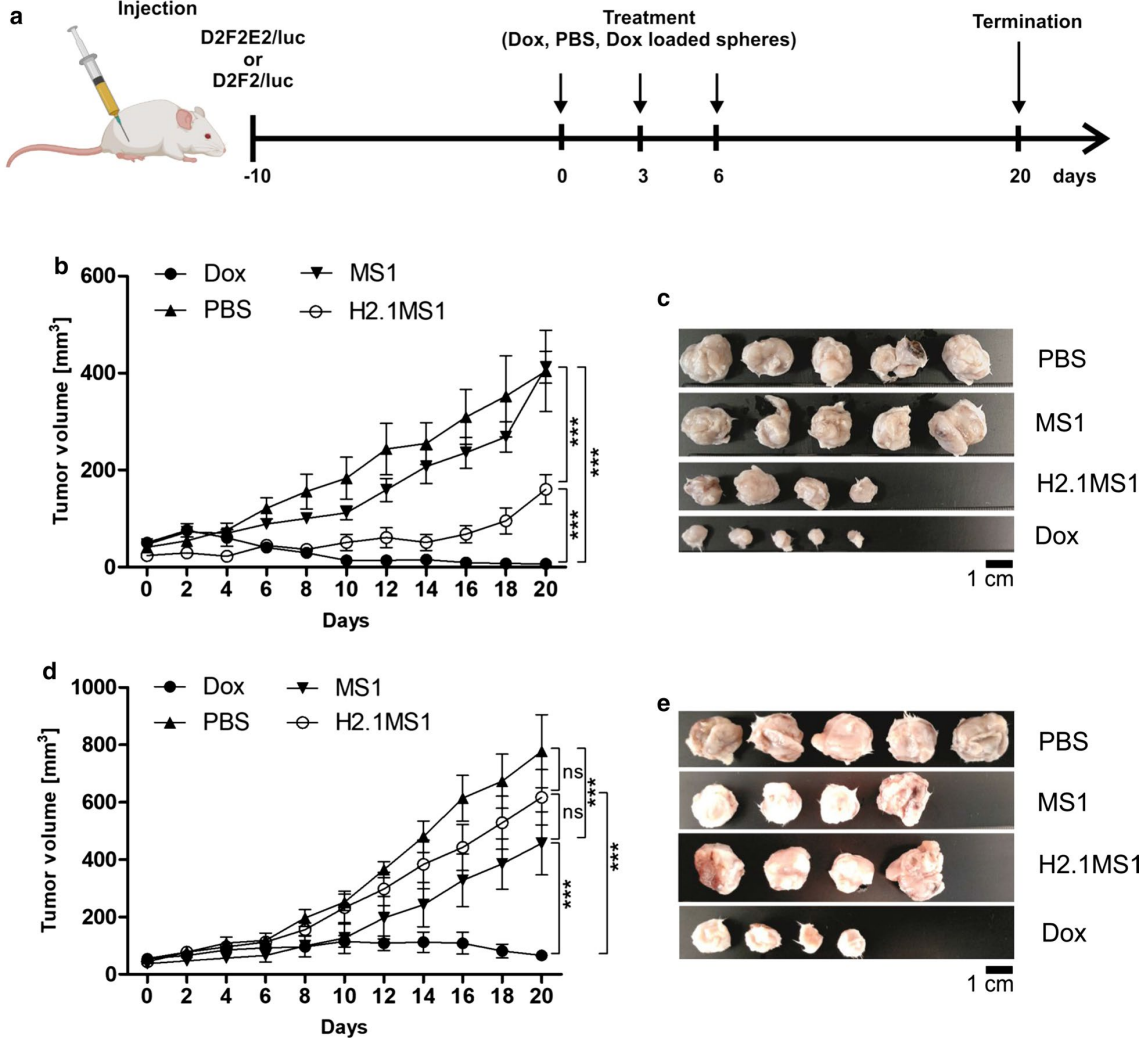
The therapeutic effect of Dox delivered in silk spheres in Her2(+) and Her2(−) mouse orthotopic breast cancer models. **a** Schematic representation of the treatment course. Mice were injected with PBS, free Dox, and Dox-loaded spheres (Dox dose = 5 mg/kg). **b** Kinetics of tumor growth during treatment in a Her2(+) breast cancer model. **c** The D2F2E2 tumors were excised 20 days after the beginning of the treatment, as indicated in **b**. **d** Kinetics of tumor growth during treatment in a Her2(−) breast cancer model. **e** The D2F2 tumors were excised 20 days after the beginning of the treatment, as indicated in **d**. The data presented are expressed as the means ± SEM; *** p < 0.001, *ns* not significant

Her2(+) and Her2(−) tumor models were used to assess the anticancer efficacy of Dox delivered by H2.1MS1 particles in an animal study. Figure 3a presents a schematic representation of the treatment. As shown in Fig. 3b, compared to the saline treatment in the control group, treatment with Dox-loaded H2.1MS1 significantly inhibited Her2(+) tumor growth. Moreover, Dox-loaded H2.1MS1 was significantly more effective in suppressing Her2(+) tumor growth than Dox-loaded MS1 with an equivalent dose of Dox. As shown in Fig. 3c, after treatment with Dox-loaded H2.1MS1 spheres, the tumors were considerably smaller than those observed after treatment with Dox-loaded control spheres or PBS.

In contrast, in control mice that developed Her2-negative tumors and received Dox-loaded H2.1MS1 particles, the mean tumor volume consistently increased until the last day of the experiment. It reached a similar mean size as that observed in mice that received PBS and Dox-loaded MS1 spheres (Fig. 3d, e).

In mice treated with free Dox, the growth of the tumors was inhibited significantly and the most effective for both Her2(+) and Her2(−) breast cancers (Fig. 3b–d).

### Assessment of the effect of the frequency of sphere administration on therapeutic efficacy

**Fig. 4:**
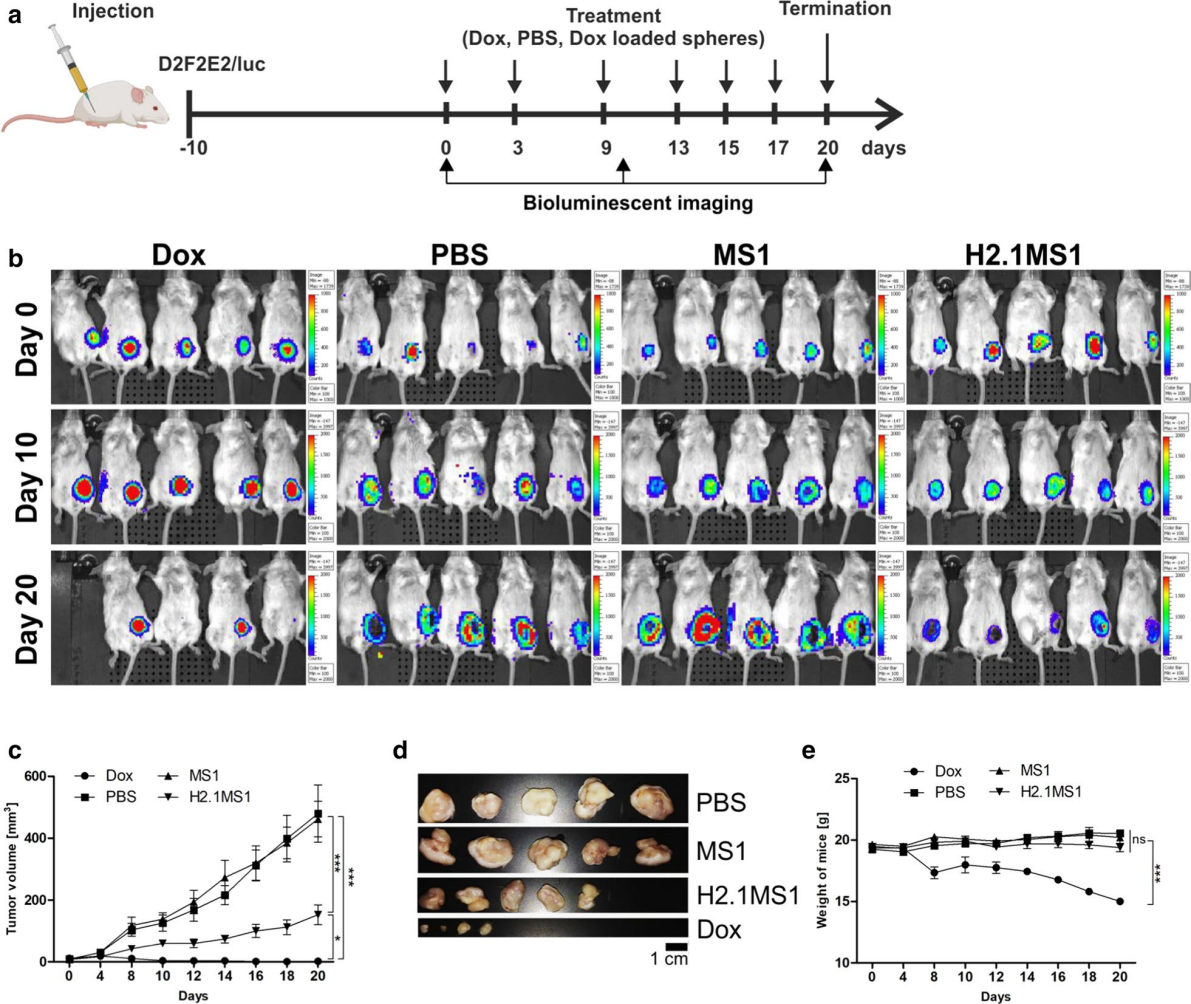
The impact of the number of treatment administrations on the therapeutic effect of Dox delivered in silk spheres in a Her2(+) orthotopic breast cancer model. **a** Schematic representation of the treatment course. Mice were injected with PBS, free Dox, and Dox-loaded spheres (Dox dose = 5 mg/kg). **b** Whole-body IVIS luminescent images of D2F2E2 tumor-bearing mice obtained using the IVIS Spectrum. **c** Kinetics of tumor growth during treatment. **d** The D2F2E2 tumors were excised 20 days after the beginning of the treatment. **e** Weight of D2F2E2 tumor-bearing mice during treatment. The data presented are expressed as the means ± SEM; *** p < 0.001, * p < 0.05

To further enhance the therapeutic effect of Dox delivered in silk spheres in a Her2(+) orthotopic breast cancer model, more frequent treatment administration was applied according to the schedule shown in Fig. 4a. The bioluminescent imaging of Her2(+) tumor-bearing mice indicated differences in tumor growth between groups that received different treatments (Fig. 4b). In mice receiving Dox-H2.1MS1 spheres, the observed luminescence intensities of the tumors were lower on days 10 and 20 compared with those of tumors at the beginning of treatment (Fig. 4b). In the control groups receiving PBS and Dox-loaded MS1 particles, a substantial increase in the luminescent signal intensity of tumors was observed as time progressed (Fig. 4b). In mice that were treated with free Dox, the number of viable tumor cells was decreased (Fig. 4b). As shown in Fig. 4c, d, in mice treated with Dox-loaded H2.1MS1 particles, the growth of the tumors was inhibited significantly compared to that observed in control groups administered PBS and Dox-loaded MS1 particles, and the excised tumors were the smallest. Moreover, the body weights were monitored during the treatment period. No significant decrease in the mean body weight in groups treated with Dox delivered by silk spheres was observed (Fig. 4e). Althought in the animals treated with the free Dox the tumor growth inhibition was the most pronounced, it was observed that the mean weight of these mice showed a significant decrease in contrast to the animals treated with Dox-loaded silk spheres at a similar drug dose (Fig. 4e). Furthermore, one animal that was treated with free Dox died.

### Analysis of the systemic toxicity of Dox carried in silk spheres

Histopathological analysis was carried out to further evaluate the possible in vivo systemic cytotoxicity of the treatment, according to the schedules indicated in Figs. 3, 4.

**Fig. 5:**
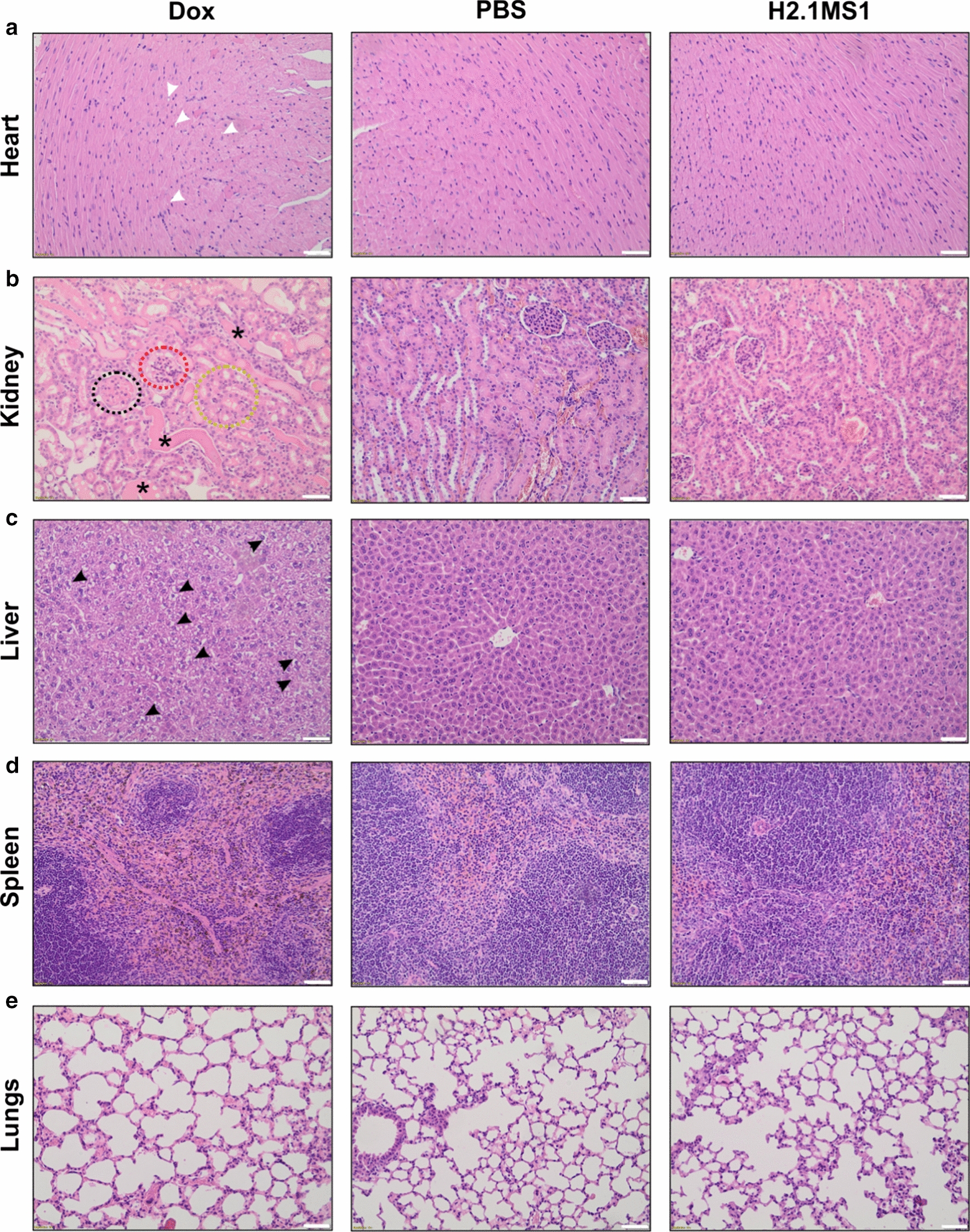
H&E staining of FFPE sections of organs collected after treatment. Her2(+) D2F2E2 tumor-bearing mice were injected intravenously with free Dox, PBS and Dox-loaded H2.1MS1 spheres according to the schedule presented in Fig. 4a. Organs such as the heart, kidney, liver, spleen, and lungs were excised on the 20th day, and the samples were stained with H&E. White arrows indicate small vacuoles in cardiomyocytes, and black arrows indicate vacuolar degeneration in the liver. The circles point out glomerular hyalinization (black), vacuolization of glomeruli (red), and hyaline droplets degeneration (yellow), and the asterisks indicate renal tubular dilatation with protein casts. Scale bar: 100 μm

The H&E-stained samples analysis did not indicate the presence of pathological changes or tumor metastasis in internal organs collected from the tumor-bearing mice after three times the administration of Dox-H2.1MS1, PBS, or Dox (Additional file 5, Figure S3). The increased number of the treatment administration (up to six times) confirmed the safety of the Dox-loaded H2.1MS1 spheres, as there were no pathological changes in the internal organs (heart, liver, spleen, lungs, and kidneys) similarly to in the saline-treated group (Fig. 5a). On the other hand, the free Dox treatment caused typical histopathologic changes [45] manifested by Dox-induced cardiotoxicity, namely vacuolar degeneration (Fig. 5a and Additional file 6: Figure S4). Additionally, hepatic lesions (hydropic degeneration and vacuolar degeneration) (Fig. 5c and Additional file 7: Figure S5), as well as nephrotoxicity (renal tubular dilatation with protein casts, glomerular hyalinization, vacuolization of glomeruli, and hyaline droplets degeneration) (Fig. 5b and Additional file 8: Figure S6) were also observed in mice receiving free Dox. Moreover, in the spleens of free Dox-treated mice, the presence of haemosiderin deposits and the prominent iron accumulation were demonstrated (Fig. 5d and Additional file 9: Figure S7). Although Dox in free drug form and encapsulated in H2.1MS1 spheres was administered at the same dose and frequency, the use of silk particles as drug carriers prevented drug-induced systemic toxicity”.

### Evaluation of the effective dose of Dox delivered by the functionalized spheres

**Fig. 6:**
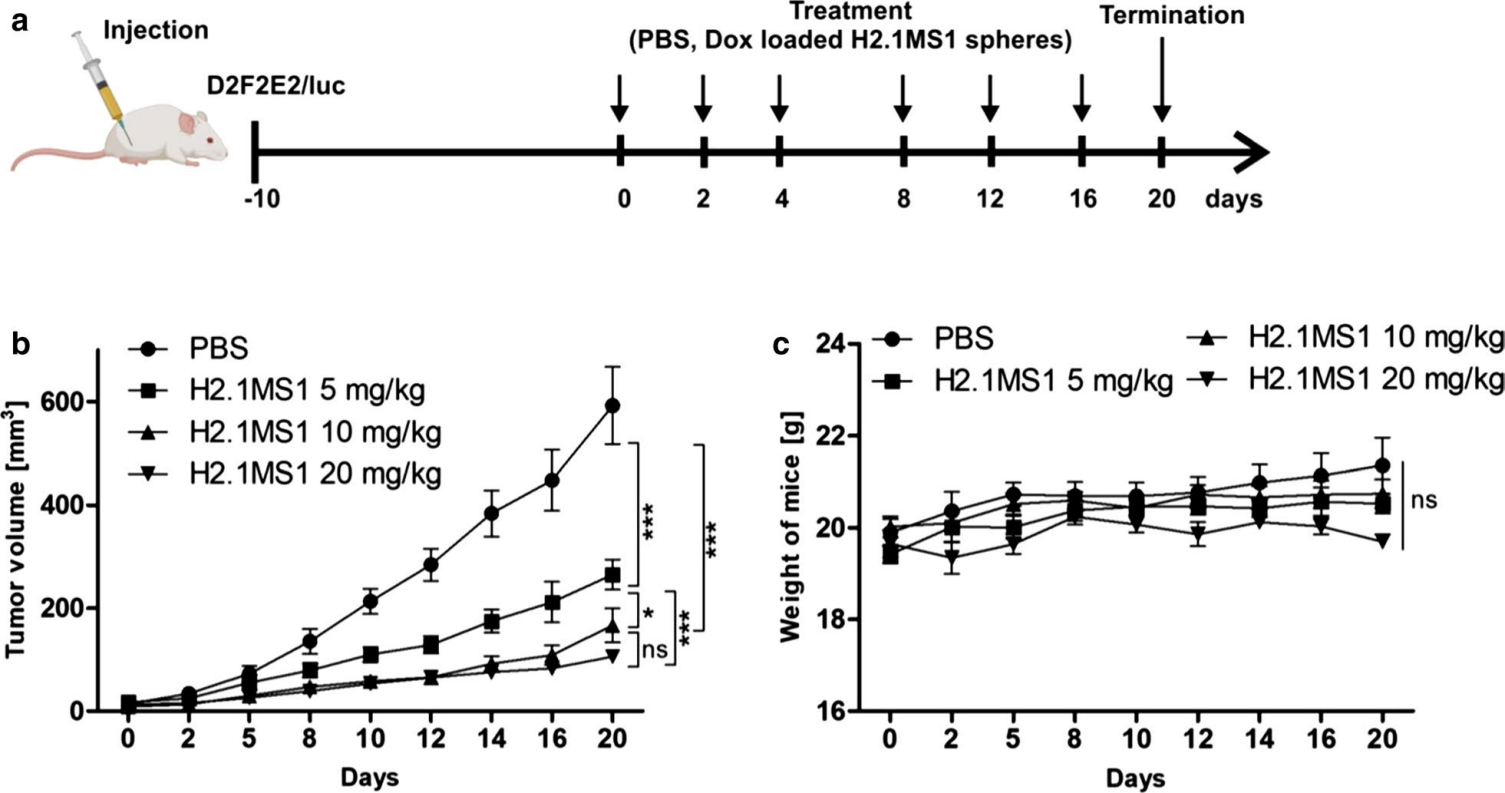
The dose-dependent therapeutic effect of Dox delivered in silk spheres in a Her2(+) breast cancer mouse model. **a** Schematic representation of the treatment course. **b** Kinetics of tumor growth during treatment. **c** Weight of D2F2E2 tumor-bearing mice during treatment. The data presented are expressed as the means ± SEM; *** p < 0.001, * p < 0.05, *ns* not significant

Next, we analyzed the effect of the drug dose on the therapeutic efficacy of Dox delivered in silk spheres in a Her2(+) orthotopic breast cancer model. Ten days after the administration of D2F2E2 cells, cancer treatment was initiated as indicated in Fig. 6a. The first dose of Dox delivered in H2.1MS1 particles was administered to three groups of mice as follows: (i) 5 mg of Dox/kg, (ii) 10 mg of Dox/kg, and (iii) 20 mg of Dox/kg b.w. The subsequent drug dosage used in the experiment was 5 mg of Dox/kg b.w for all treated groups of animals. The control mice received PBS.

In all groups treated with Dox-loaded H2.1MS1 spheres, independent of the dose applied, significant tumor growth inhibition was observed compared to that in PBS-treated mice (Fig. 6b). There was a significant difference between animals initially treated with 10 and 20 mg of Dox/kg b.w. and mice who received a lower dosage of 5 mg of Dox/kg b.w. for the first dose (Fig. 6b). Moreover, the analysis of tumors excised from animals after the treatment confirmed that the smallest tumors were found in a group of mice that was administered a dosage of 20 mg of Dox/kg b.w. (Additional file 10: Figure S8).

In contrast, in mice bearing Her2(−) breast cancers, no effect of the dosage of the first drug dose on the treatment efficacy was observed (Additional file 11: Figure S9a–b). For all treatments, independent of the applied drug dose, mice did not lose weight, which implied that the examined formulations were not toxic (Fig. 6c and Additional file 11: Fig. 9c).

### Histological analysis of orthotopic breast tumors after treatment

**Fig. 7:**
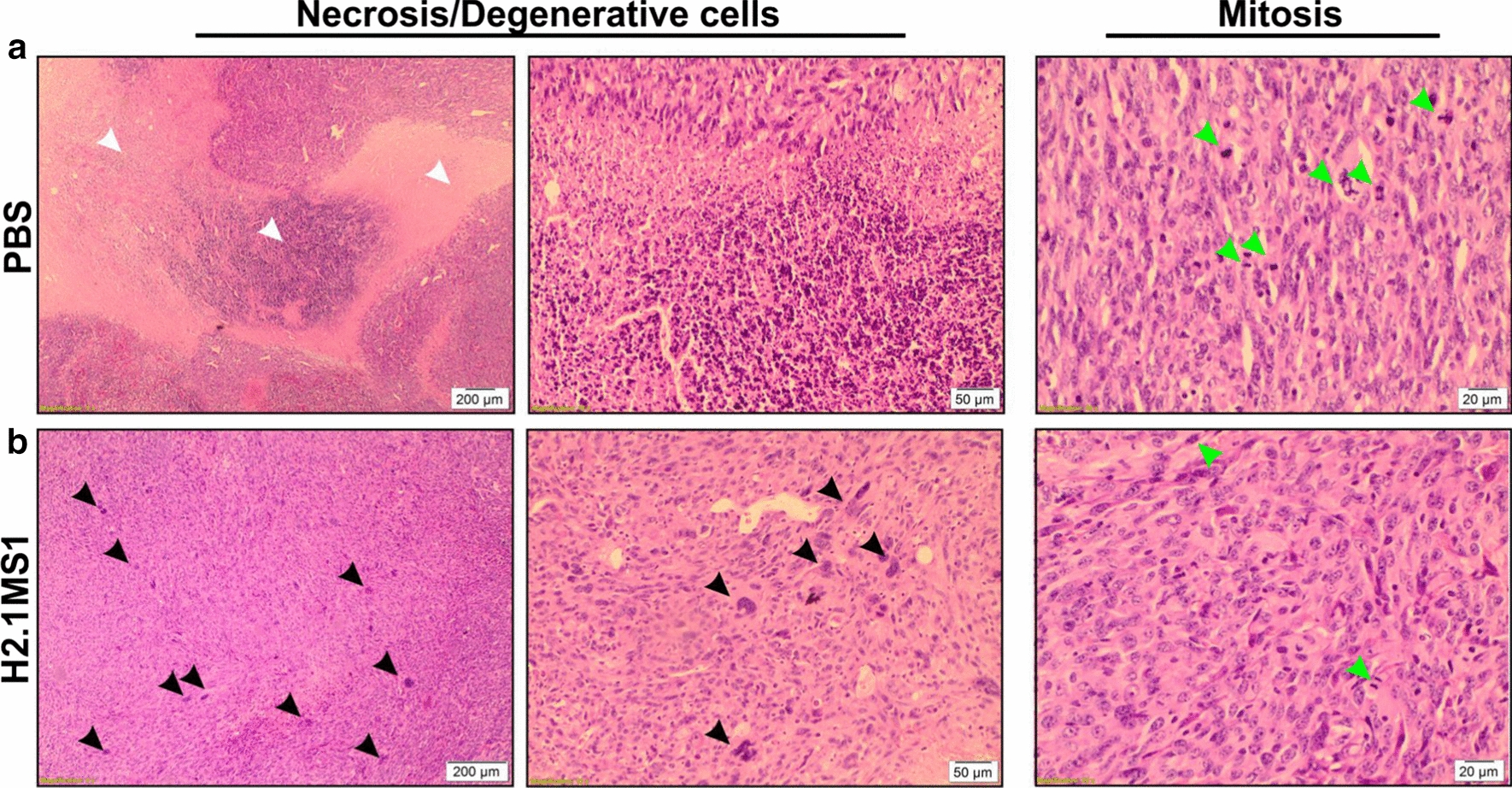
Histological analysis of tumors that developed in an orthotopic breast cancer model. H&E staining of tumor sections from D2F2E2 tumor-bearing mice after 20 days of treatment with **a** PBS and **b** 20 mg of Dox/kg b.w. loaded in H2.1MS1 particles, according to the schedule presented in Fig. 5a. On the left and in the middle: analysis of necrotic and degenerative cells at two different magnifications, 4X and 10X, respectively. On the right: analysis of mitosis. Black arrows indicate degenerative cells; white arrows—necrosis; green arrows—mitosis. Scale bars: 200 µm, 50 µm, and 20 µm

After 20 days of the different treatments, the Her2(+) tumor-bearing mice treated with Dox-loaded H2.1MS1 particles at a dosage of 20 mg of Dox/kg b.w. exhibited the most prominent antitumor effect (Fig. 6b and Additional file 10: Figure S8). The histological evaluation of tumors treated with PBS indicated the high proliferative activity, as indicated by a large number of mitotic cells and massive necrosis (Fig. 7a). In contrast, a higher number of degenerative cells in tumor tissue from Dox-loaded H2.1MS1-treated mice than in tumor tissue from PBS-treated mice was observed (Fig. 7b).

### Biodistribution of silk spheres in a model of metastatic breast cancer

**Fig. 8:**
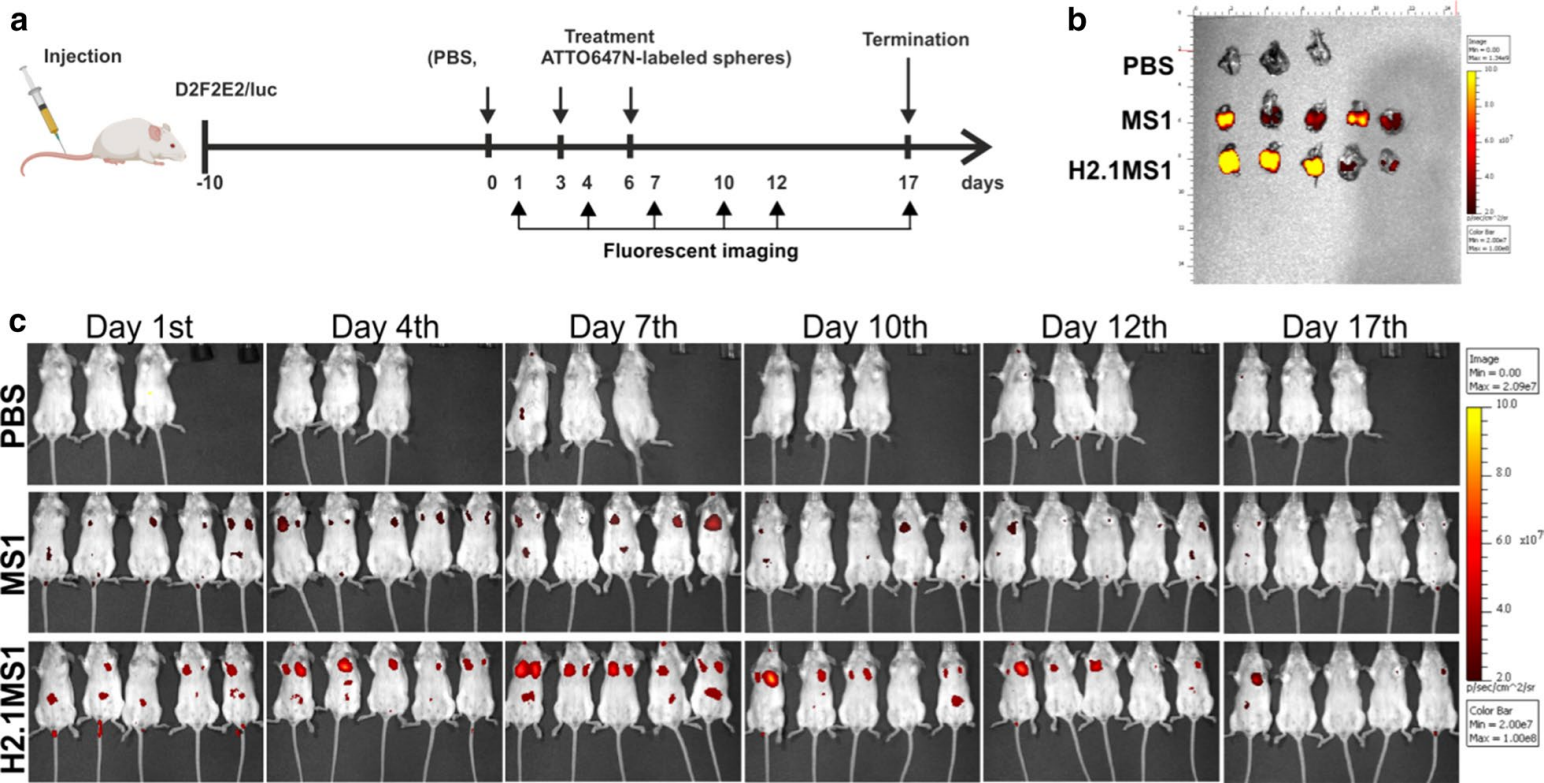
Biodistribution of silk spheres in a Her2(+) model of metastatic breast cancer. **a** Schematic representation of the treatment course. **b** Biodistribution of the control MS1 and H2.1MS1 spheres in the lungs on the 17th day after the first intravenous injection. The lungs were excised and imaged. Signal detection was performed by using the IVIS Spectrum at excitation/emission wavelengths of 640/680 nm. **c** Biodistribution of ATTO647N-labeled MS1 and H2.1MS1 spheres over time. Whole-body IVIS fluorescent images were obtained using the IVIS Spectrum

A mouse model of Her2(+) tumor metastasis was established by injection of D2F2E2 cells via the tail vein into BALB/c mice. In this model, mice were treated according to the schedule presented in Fig. 8a. Twenty-four hours after the second injection (at day 4), a notable accumulation of H2.1MS1 particles was found in the lungs (Fig. 8c). The accumulation of the control MS1 spheres in the lungs after intravenous administration was substantially lower than that of the functionalized H2.1MS1 particles (Fig. 8c). Seventeen days after the first sphere injection, the lungs were collected and imaged using an IVIS imaging system. The H2.1MS1 and MS1 particles were detectable in the excised lungs (Fig. 8b). All mice in this experiment developed Her2(+) D2F2E2 metastases in the lungs, as indicated in Additional file 12, Figure S10.

### Antitumor efficacy of Dox-loaded functionalized silk spheres in a model of metastatic breast cancer

**Fig. 9:**
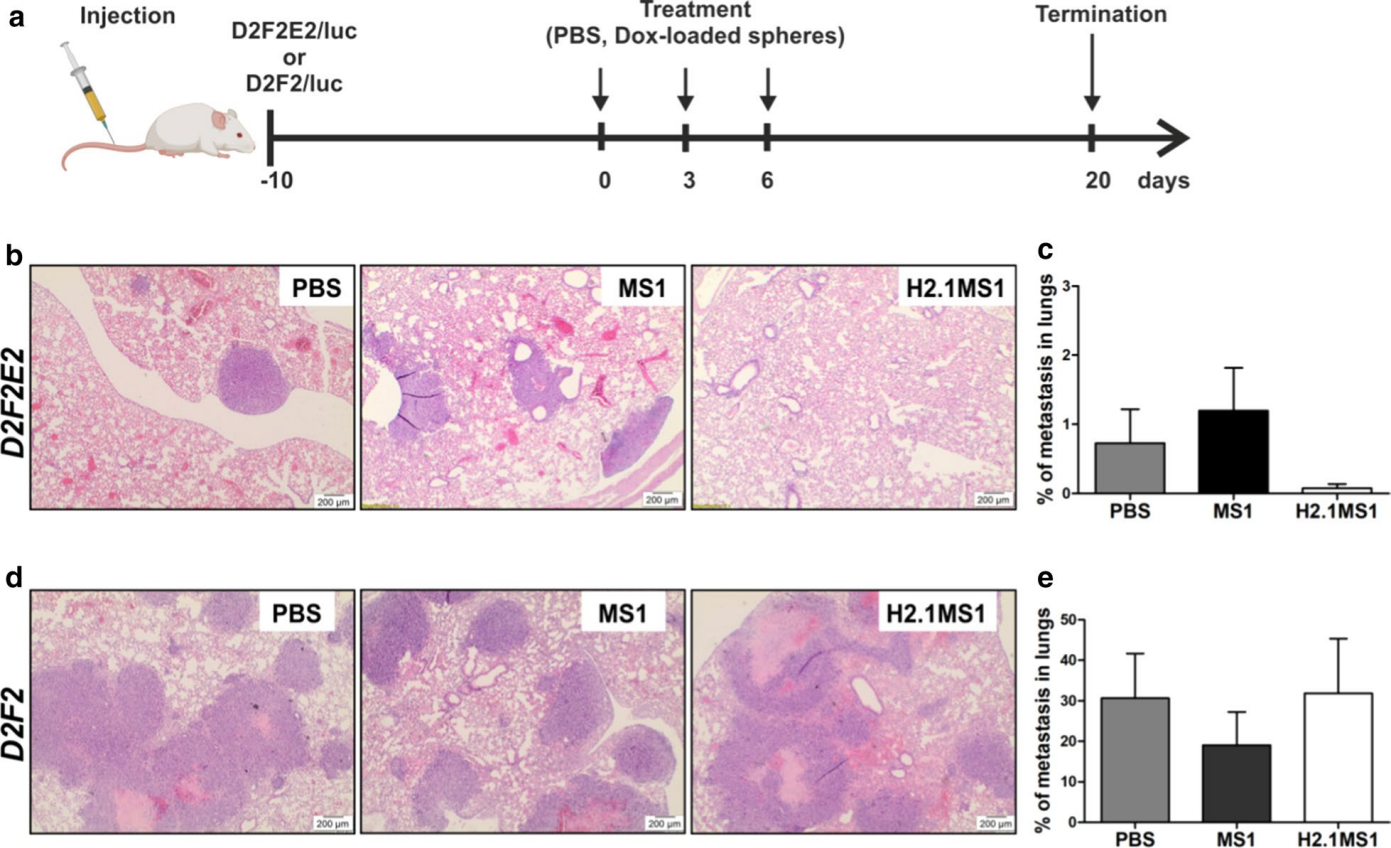
The therapeutic effect of Dox delivered in silk spheres in Her2(+) and Her2(−) breast cancer models of metastasis. **a** Schematic representation of the treatment course. The histopathological analysis of the lung tissue sections stained with H&E from **b** D2F2E2 and **d** D2F2 tumor metastasis models after  20 days of treatment. Scale bar: 200 µm. The percentage of lung tissue affected by **c** D2F2E2 and **e** D2F2 tumor metastasis. The data are presented as the means of 3 specimens ± SD

Her2(+) and Her2(−) breast cancer models of metastasis were used to further evaluate the antitumor efficacy of Dox delivered by silk particles. Figure 9a presents a schematic of the course of treatment.

Mice treated with Dox-loaded H2.1MS1 particles exhibited a prominent antitumor effect in the Her2(+) tumor model (Fig. 9, Table 1). As shown in the histological analysis of lung tissue sections, treatment with the Dox-loaded H2.1MS1 spheres substantially suppressed Her2(+) metastasis growth compared to treatment with the Dox-loaded MS1 spheres and saline (Fig. 9b, c, Table 1). As shown in Table 1 and Fig. 9c, in mice treated with Dox-loaded H2.1MS1 spheres, the percentage of lung tissue affected by metastasis (metastasis index) was considerably smaller (at least 10 times) than that in mice treated with Dox-loaded control spheres or PBS. To strengthen our results, the samples were analyzed using histopathological evaluation. However, the metastasis observed in samples obtained from Dox-H2.1MS1-treated mice was so rare and minimal that it was not possible to adjust the histopathological standards for quantitative analysis (Table 1, Additional file 13: Table S2).

**Table 1:** Histopathological analysis of tumors developed in a metastasis model of breast cancer

	D2F2E2			D2F2		
	PBS	MS1	H2.1MS1	PBS	MS1	H2.1MS1
Metastasis index	0.730 (± 0.848)	1.197 (± 1.068)	0.078 (± 0.106)	30.653 (± 19.09)	19.063 (± 14.236)	31.867 (± 23.336)
Number of mitoses (5HPF-40X)	26 (± 4)	11 (± 2) *	ND	46 (± 13)	34 (± 18)	26 (± 11)
Degree of apoptosis	1	1	ND	2	1.66 (± 0.58)	2.3 (± 0.58)
Necrosis index	0	0	ND	8.983 (± 2.309)	1.083 *** (± 0.553)	5.197 (± 4.375)
% of degenerative cells	0.533 (± 0.681)	4.533 (± 3.579)	ND	0.167 (± 0.289)	5.933 (± 5.198)	4.733 (± 4.248)

H&E-stained lung tissue sections from mice injected with Her2(+) cells and Her2(−) cells were characterized according to the histopathological classification. The data are presented as the means of 3 specimens ± SD; *** p < 0.001, * p < 0.05

*ND* not determined

The analysis of the randomly selected sample of the Her2-positive tumors treated with Dox-loaded H2.1MS1 spheres indicated the considerably reduced proliferative activity of tumor cells; lower expression of the proliferation marker Ki-67 was observed in this sample when compared with that in the Dox-loaded MS1- and saline-treated samples (Additional file 14: Figure S11). TUNEL staining of the apoptotic cells indicated a similar number of TUNEL-positive cells in all treated groups (Additional file 15: Figure S12).

In control mice that developed Her2-negative tumor metastasis and received Dox-loaded H2.1MS1 particles, the mean metastasis index reached a similar value as that observed in mice that received PBS and Dox-loaded MS1 spheres (Fig. 9d, e, Table 1). Moreover, a histological analysis indicated the large-scale presence of necrotic tissue in metastatic foci independent of the applied treatment (Fig. 9d, Table 1). The IHC analysis of the Ki-67 marker indicated a similar level of expression in all treated groups (Additional file 14: Figure S11). Additionally, the apoptosis score, which was based on the number of TUNEL-positive cells, was equal for all tested Her2(−) samples (Additional file 15: Figure S12).

The raw data used for the histopathological assessment of tumors in the model of breast cancer metastasis are included in the Supplementary Material (Additional files 13 and 16: Table S2 and Table S3, respectively).

In general, Her2(−) breast cancer cells developed into larger tumors in mice than Her2(+) cells. The larger sizes of D2F2 tumors were macro- and microscopically noticeable in both orthotopic (Fig. 3, and Additional file 11: Figure S9) and metastatic (Fig. 9, Table 1, and Additional file 17: Figure S13) models.

## Discussion

In this work, we evaluated the potential of functionalized silk nanocarriers (spheres) for targeted drug delivery in vivo. We found that spheres functionalized with a peptide that provides specific cell recognition and intracellular internalization significantly enhanced the therapeutic effect of the drug delivered to cancer cells. The therapy did not cause any toxic side effects in mice. Moreover, the experiments were performed in immunocompetent mice and did not impede the efficient delivery of the drug by the H2.1MS1 spheres, which exerted a therapeutic effect.

In our previous work, we developed a targeted drug delivery system (DDS) in vitro. Our system was based on MS1 silk spheres that were functionalized with the H2.1 peptide, which recognizes the Her2 molecule. The H2.1MS1 particles efficiently delivered doxorubicin to the cells overexpressing Her2, which induced significantly greater cytotoxicity in Her2(+) cancer cells compared with that induced in other cell lines without Her2 overexpression and by control spheres without functionalization [40].

To examine the potential benefit of using the obtained DDS, in vivo mouse models of breast cancer were developed by using D2F2 and D2F2E2 cell lines with genetically induced overexpression of Her2 [46]. First, we found in vitro that by using these cells and our DDS, we obtained a similar therapeutic effect as that observed in our previous studies when human breast cancer cells were applied [40]. Indeed, the H2.1MS1 spheres bound significantly more to D2F2E2 cells and efficiently delivered drug to these cells than the control particles (without functionalization). In addition, the H2.1MS1 spheres did not deliver drug to control D2F2 cells. Both particle types (functionalized and control) were similar in terms of morphology and drug loading efficiency.

We developed two mouse models of breast cancer that mimicked primary and metastatic lesions. To obtain the primary tumor model, we administered the cancer cells into the mammary pad (the orthotopic model), and for metastasis formation, we administered cancer cells via the tail vein. After the injection of cancer cells into the tail vein, metastases are formed in the lungs [47–49], which we confirmed histopathologically in our model.

The biodistribution study of the DDS indicated that the location of the tumor lesion influenced the accumulation of the spheres. When Her2(+) breast cancer developed in a mammary pad, the intravenously administered H2.1MS1 spheres accumulated in the tumor lesion, which was in contrast to what was observed in the model when Her2(−) cancer cells were applied. This could indicate that the Her2-H2.1MS1 interaction was crucial to sustaining the localization of the particles.

On the other hand, in the Her2-overexpressing metastasis model, both types of spheres (functionalized and control) accumulated in the lungs. In general, after intravenous injection of the DDS, the particles were initially directed to the lungs [25, 50, 51]. As shown by in vivo imaging, in both tumor models (primary and metastatic), we initially observed the signal from the silk spheres in a location that indicated the lungs. However, in the primary tumor model, sphere clearance from the lungs was much faster than that in the metastasis model. The data obtained from the excised organs confirmed that in the metastasis model, 20 days after the first sphere administration, both sphere types were observed in the lungs. The accumulation of both types of particles in the lungs could result from passive tumor targeting, which takes advantage of the EPR effect. Due to the administration method used, a much higher concentration of particles first appeared in the lungs. This could contribute to the enhancement of the EPR effect for both the H2.1MS1 and MS1 spheres. In contrast, a lower concentration of particles could be delivered to the tumors in the mammary pad; thus, only the specific accumulation of spheres was observed. However, this needs further study.

In the primary breast cancer model, the results indicated that the Her2-H2.1MS1 interaction exerted a significant effect on doxorubicin cytotoxicity. Independent of the frequency of sphere administration, H2.1MS1 particles were more effective in the delivery of the active form of doxorubicin than control MS1 spheres in the Her2(+) tumor model. We observed that the frequency and dose of the delivered drug were crucial for more effective cancer treatment. When the administration of the spheres was interrupted for more than two or three days, the tumor size increased. Further study is needed of the number and dosage of drug-loaded spheres required to ensure complete tumor destruction. Furthermore, other approaches can be investigated. Recently, w have proposed a silk-based DDS that, from one side, demonstrated the control of loading and release of Dox in the silk-spheres and, from the other side, targeted drug delivery [52]. The double functionalized silk spheres were formed by blending two silks: H2.1MS1 and DOXMS2. The new silk DOXMS2 was constructed and functionalized with DOX peptide of an affinity to doxorubicin [52]. These particles showed superior doxorubicin-loading capacity and specific binding per cell, with simultaneously low Dox release at a pH of 7.4. Such property may prevent the drug from being released into circulation before the particles reach the tumor. The in vivo application of these spheres may further improve the silk-based DDS efficiency.

In the D2F2E2 metastasis model, our preliminary data indicated that the average surface area of the metastases treated with the drug-loaded H2.1MS1 particles was at least 10 times smaller than that observed in the control groups. Although we did not observe a significant difference, the trend was evident. A more extensive study (with more animals per group) is needed to verify this data. Moreover, the results obtained in the D2F2 metastasis model confirmed that the Her2–H2.1MS1 interaction was necessary to treat cancer efficiently. No therapeutic outcome was observed in the Her2(−) model.

On the other hand, both types of spheres (functionalized and control) that accumulated in the lungs (probably in the tumor lesions) could release doxorubicin in the tumor microenvironment (TME). Indeed, a trend toward an increase in the number of degenerative cells and a reduction in the number of mitotic cells was observed in these tumor samples. Moreover, Her2(−) cancer cells formed larger tumors in mice than Her2(+) cells. The size, metastasis index, and necrosis index were considerably larger for Her2-negative tumors than for Her2-positive tumors. In larger tumors, particle accumulation in the TME due to the EPR effect could exert a greater effect. Thus, some therapeutic symptoms due to D2F2 cancer could be observed even when Dox-loaded MS1 spheres were applied. However, drug release from the control spheres into the TME was not sufficient to treat cancer.

We previously showed in vitro that the binding of H2.1MS1 particles to Her2 initiated the endocytosis process, and spheres were transported to endosomes and then to lysosomes [44]. The activity of the enzymatic environment in the lysosomes was necessary for sphere degradation [44]. Moreover, doxorubicin delivered intracellularly (via H2.1MS1 particles) was significantly more efficient in killing the cells than doxorubicin released in the environment from control MS1 particles [40]. Previously, it was shown that Dox-loaded silk spheres released the drug in a pH-dependent manner (faster at an acidic pH and slower at a neutral pH) [40, 53]. Many Dox carriers take advantage of the acidic pH of the TME to induce the release doxorubicin [53–56]. Although we observed the accumulation of both types of spheres in the lungs in the metastasis model, only the interaction of D2F2E2-Dox-loaded H2.1MS1 resulted in a severe reduction in metastasis. This result indicated that the targeted drug delivery system that produces internalization of the drug carrier is much more effective than a DDS that allows for the accumulation of drug vehicles in the TME. Changing the Dox distribution so that it favors the intracellular versus the extracellular compartment of the tumor is essential to induce pharmacodynamic changes that improve the therapeutic efficacy [57]. Accordingly, targeting mediated by the Her2 ligand might not only quantitatively enhance drug localization in the TME but also qualitatively change the drug delivery mechanism in tumor cells to increase efficacy. Carriers that combine passive and active drug delivery have great potential for cancer treatment.

The studies were performed in immunocompetent mice. The cancer cells injected into the mice originated from the same mouse strain [46, 58]. The administration of silk spheres (with or without Dox) did not produce side effects, regardless of the type of silk spheres used or the frequency or dose of their application. Although the spheres were partially localized in the liver and lungs, the histopathological analysis did not reveal any adverse effects related to silk particle accumulation. Moreover, the administration of the silk spheres did not cause weight loss in mice, in contrast to the administration of free doxorubicin. This indicates that the drug delivery system based on silk utilizes a promising biomaterial for in vivo applications. However, its toxicity and potential immunogenicity requires more advanced studies.

## Conclusion

To the best of our knowledge, for the first time, we demonstrated the effectiveness of bioengineered silk spheres for targeted drug delivery for cancer treatment in in vivo models. Cellular recognition and sphere internalization were crucial to ensure the significant toxic effect of the delivered drug. The administration of different numbers of spheres and increased dosages of spheres did not cause any macro- and microscopic side effects. Moreover, the studies were performed in mice with an active immunological system. In summary, we revealed that functionalized silk spheres represent a promising tool as a targeted drug delivery system.

## Materials and methods

### Reagents

Doxorubicin hydrochloride (DOX-HCl, Dox) was purchased from Pfizer Inc. (Adriamycin, Pfizer Inc., New York, NY, USA). Herceptin, an anti-Her2 antibody, was obtained from Roche (Trastuzumab, Roche, Basel, Switzerland). The herceptin and silk proteins were labeled with the ATTO647N fluorophore (Sigma, St. Louis, MO, USA) according to the manufacturer’s protocol.

### Expression and purification of bioengineered spider silks

The MS1 and H2.1MS1 proteins were designed, expressed, and purified as described previously [40].

### Silk sphere preparation

The MS1 and H2.1MS1 silk solutions at a concentration of 0.5 mg/mL were sterilized using a 0.22 µm filter and then mixed with sterile 2 M potassium phosphate buffer, pH 8.0, under aseptic conditions (Sigma, St. Louis, MO, USA) at a volumetric ratio of 1:10 (100:1000 μL). For silk sphere preparation, a micromixing technique with a syringe pump system (neMESYS 2600 N, Cetoni GmbH, Korbussen, Germany) was implemented as described previously [41]. The resulting particles were incubated at room temperature for 12 h. The suspensions were dialyzed against ultrapure sterilized water and centrifuged at 10 000 g for 30 min. The spheres were redispersed in autoclaved water. The concentration of spheres was determined gravimetrically. Spheres were loaded with the drug as indicated in the Additional files.

### Cells

D2F2 and Her2-overexpressing D2F2E2 murine breast cancer cell lines derived from the female BALB/c mouse strain were a kind gift from Prof. Constantin Baxevanis (Cancer Immunology and Immunotherapy Center, Saint Savas Cancer Hospital, Athens, Greece). D2F2E2 cells were obtained by modification of D2F2 cells with a vector expressing the human Her2/*neu* gene [46]. D2F2E2 cells required the presence of geneticin in the culture medium (G-418, 200 µg/mL, Sigma, St. Louis, MO, USA) to maintain Her2 expression. For luminescence imaging, both cell lines were modified by the incorporation of cDNA encoding a luciferase (LUC). D2F2/LUC cells were modified with pGL4.51-Luc (Promega, Madison, WI, USA), and D2F2E2/LUC cells were generated by transfection with pcDNA3.1-Luc (Addgene, Watertown, MA, USA) using Lipofectamine LTX PLUS Reagent (Invitrogen, Carlsbad, CA, USA). The D2F2/LUC and D2F2E2/LUC cells were selected to obtain stable clones and characterized as described in the Supplementary section. The cells were routinely grown in Dulbecco’s modified Eagle medium (DMEM, Biowest, Nuaillé, France) containing 10% fetal bovine serum (Biowest, Nuaillé, France) and 80 µg/mL gentamycin (KRKA, Novo Mesto, Slovenia) in a humidified atmosphere of 5% CO_2_ at 37 °C.

### Cytotoxicity study by MTT assay

For cytotoxicity study, a total of 2.5 × 10^4^ of cells D2F2E2/LUC and D2F2/LUC per well were seeded onto a 96-well plate 24 h before the cytotoxicity experiment. Next, different concentrations of functionalized or control silk spheres loaded with Dox were added to the cell cultures as indicated. After 4 h of incubation, cells were washed with PBS, and fresh medium was added. The cells treated with medium without spheres were used as a negative control. After 72 h of incubation, the mitochondrial activity of cells was assessed using MTT assay, as described previously [40]. The relative cell viability (%) related to the negative control was calculated by using the following equation:

$$\left(\text{test sample}\right)/(\text{negative control}) \times 100{\%}$$

The experiments were repeated three times in triplicate.

### Cellular uptake of silk spheres analyzed by flow cytometry (FCM)

D2F2E2/LUC and D2F2/LUC cells were washed with PBS/0.5% BSA and detached with nonenzymatic cell dissociation solution (Sigma, St. Louis, MO). Next, the ATTO647N-labeled spheres were added at a final concentration of 10 µg/mL to 1 × 10^5^ cells suspended in PBS/0.5% BSA and incubated for 1 h at 4 °C in the dark. After washing three times with PBS, the fluorescence data were collected in the FL4 channel of the BD FACSAria flow cytometer (BD Pharmingen, San Jose, CA, USA) and analyzed using FlowJo software (Tree Star, Ashland, OR, USA). Three independent experiments were performed.

### Doxorubicin incorporation into spheres

To load the Dox into the MS1 and H2.1MS1 silk spheres a 50 µg of silk particles were suspended in 250 µL PBS pH 7.4 and then mixed with 50 µL of Dox at a concentration of 2 mg/mL to get a final volume of 300 µL. The solution was incubated overnight at room temperature under continuous shaking. After 12 h of incubation, the spheres were centrifuged, and the drug concentration in the supernatant was determined spectrophotometrically using a UV1600PC spectrophotometer (VWR Ltd., Radnor, PA, USA). For Dox measurements, a wavelength of 509 nm was applied. A standard calibration curve of Dox was used for drug quantification. The total amount of the drug-loaded into the silk spheres was calculated by subtracting the amount of the drug remaining in the supernatant from the amount added initially. The encapsulation efficiency was calculated using the following equation:

$$\left(\text{amount of Dox in the sample}\right)/(\text{amount of drug initially added}) \times 100\mathrm{\%}$$

### Animals

Six-week-old female BALB/cAnNCrl mice were purchased from Charles River Laboratories International, Inc. (Erkrath, Germany). The animals were maintained under constant pathogen-free conditions with a 12 h light/dark cycle and water and food provided ad libitum. Mice were used at the age of 9–10 weeks. All experiments were performed according to the national and institutional guidelines for the humane treatment of laboratory animals after approval by the Local Ethical Committee for Experiments on Animals in Poznan, Poland (No 35/2014, 34/2017, and 72/2017). All efforts were made to minimize animal suffering.

### Molecular imaging

Bioluminescence images of tumors were captured using the IVIS^®^ Spectrum in vivo imaging system (Perkin Elmer, Santa Clara, CA, USA) and analyzed using Living Image IVIS^®^ imaging software (Perkin Elmer, Santa Clara, CA, USA). When needed, ten minutes before imaging, animals received an intraperitoneal injection of 200 µL of D-luciferin sodium salt (15 mg/mL; StayBrite, Biovision, Milpitas, CA, USA) prepared in PBS. Anesthesia was maintained with inhaled 2% isoflurane (Forane, Abbott Laboratories, North Chicago, IL, USA) using an XGI 8 gas anesthesia system (Xenogen, Perkin Elmer, Santa Clara, CA, USA). Animals were positioned on the IVIS warming stage in the dorsal position. The default bioluminescent settings of Living Image were used with a scanning time of 10 s and with the F/stop adjusted to prevent image saturation. The in vivo imaging of cancers was performed according to the indicated schedule.

The biodistribution of the ATTO647N-labeled silk spheres was assessed with the IVIS Spectrum system using 640 nm excitation and 680 nm emission wavelengths. The fluorescent imaging of silk particles was performed according to the indicated schedule.

### Mouse orthotopic model of breast cancer

Female 9–10-week-old BALB/cAnNCrl mice received 1 × 10^6^ D2F2E2/LUC or D2F2/LUC cells to induce the development of cancer. The cells were washed and harvested in PBS, and then 0.1 mL of cell suspension was injected into the mammary fat pad of the mouse. The sphere biodistribution studies or the cancer treatment were initiated when the tumor volume reached approximately 50–100 mm^3^, which was recorded as day 0 (typically on the 10th day after the administration of the cells), and studies were conducted as indicated. On the last day, mice were sacrificed by using a device designed for the gas euthanasia of small rodents (TEM-SEGA, Pessac, France), and the tumors and organs were excised for further analysis.

#### Biodistribution of the silk spheres in an orthotopic model of breast cancer

Preliminary research was conducted by using individual animals to analyze the biodistribution of the silk particles in a mouse model of orthotopic breast cancer. D2F2E2/LUC or D2F2/LUC tumor-bearing mice were treated intravenously with 150 µg of ATTO647N-conjugated H2.1MS1 particles. The mice received the tested spheres three times via retro-orbital venous sinus injection on days 0, 3, and 6. Moreover, the control animals received 5 µg of ATTO647N-labeled Herceptin intravenously. The level of fluorescent signal accumulation in the tumor and/or organs was evaluated by whole body in vivo imaging (24 h after each sphere administration) or ex vivo imaging (48 h after the 3rd sphere injection) using the IVIS Spectrum system and Living Image software.

#### The therapeutic effect of Dox delivered in silk spheres in an orthotopic breast cancer model

The D2F2E2/LUC or D2F2/LUC tumor-bearing mice were divided into the following groups that received intravenous (i) PBS, (ii) free Dox (5 mg/kg b.w.), (iii) Dox-loaded MS1 spheres (corresponding to 5 mg of Dox/kg b.w.), or iv) Dox-loaded H2.1MS1 spheres (corresponding to 5 mg of Dox/kg b.w.). Each group contained at least 4 animals. To ensure a dose of 5 mg Dox per kg b.w was received, approximately 150 µg of Dox-loaded spheres were administered. The mice received treatments according to various schedules as indicated. The development of the tumors was evaluated using the IVIS Spectrum system on days 0, 10, and 20 according to the luminescence signal of cancer cells. Moreover, the tumors were measured every two days by using a caliper, and the tumor volumes (V) were calculated as follows:

$$V=L\times {W}^{2}/2$$

where L is the tumor length and W is the tumor width. Moreover, the body weight of the mice was routinely measured, and the mice were monitored for any adverse health effects. To analyze the therapeutic effect of the dose of Dox delivered in spheres, the mice received different amounts of Dox-loaded H2.1MS1 spheres corresponding to (i) 5 mg of Dox/kg b.w. (n = 8), (ii) 10 mg/kg b.w, (n = 8), and (iii) 20 mg/kg b.w. (n = 8) on day 0, and the subsequent dosages corresponded to 5 mg/kg b.w. On day 20, the mice were sacrificed, and the tumors were excised for further analysis.

### Mouse model of metastatic breast cancer

Nine- to ten-week-old BALB/cAnNCrl female mice received intravenous injections via the tail vein of 3 × 10^5^ D2F2E2/LUC or D2F2/LUC cells. The analysis of the biodistribution of the spheres or cancer treatment started 10 days after tumor cell administration. All animals were sacrificed by using gas euthanasia 17 or 20 days after the injection of tumor cells, and the lungs were excised for further analysis. The presence of metastatic nodules in the lungs was confirmed by histological analysis.

####  Biodistribution of the silk spheres in a metastatic model of breast cancer

BALB/cAnNCrl female mice were injected with D2F2E2/LUC cells to induce metastasis formation. Ten days after the injection of the cells, the animals were divided into three groups and treated intravenously with ATTO647N-conjugated control MS1 spheres (n = 5), functionalized H2.1MS1 particles (n = 5), and PBS (n = 3). The mice received 150 µg of the tested formulations three times via retro-orbital venous sinus injection on days 0, 3, and 6. The level of sphere accumulation in the organs was evaluated according to the fluorescence of the particles and whole body IVIS fluorescent imaging. On day 17, the animals were sacrificed, the lungs were collected, and then the sphere fluorescence was examined ex vivo using the IVIS Spectrum.

####  The therapeutic effect of Dox delivered in silk spheres in a metastatic model of breast cancer

Ten days after the injection of D2F2E2/LUC or D2F2/LUC tumor cells, BALB/cAnNCrl female mice received three intravenous injections via the retro-orbital venous sinus of the drug-loaded MS1 or H2.1MS1 spheres at a dosage corresponding to 5 mg of Dox/kg b.w. Control animals received saline. Each group contained 3 animals. On day 20, the animals were sacrificed, and the lungs were collected for further analysis. Before lung collection, these organs were injected with 1.2 ml of 10% neutral buffered formalin (Amresco, Solon, OH, USA) by tracheal cannulation to fix the inner airspaces and inflate the lung lobes.

### Histological analysis

Tumors or internal organs, such as the heart, kidneys, lungs, and liver, were excised from the mice as indicated, fixed with 10% formalin, and then used for routine histopathological processing. The sections were automatically stained using a standard histological protocol with H&E (Leica Biosystems, Wetzlar, Germany). Samples were visualized under a light microscope (BX53, Olympus Corporation, Tokyo, Japan) and imaged using a microscope mounted digital camera and the program CellSens (Olympus Corporation, Center Valley, PA, USA). The morphometric analysis was performed by using the ImageJ 1.51 K program (Wayne Rasband, National Institute of Health, USA).

To assess treatment-induced systemic toxicity, samples were analyzed in terms of the macroscopic changes in the examined organ sections, including the presence of inflammatory infiltrating cells and necrotic foci in organs, the presence of edema in the alveoli and interstitial lung fibrosis, the assessment of myocardial cell size, cardiac wall thickness, and the presence of vacuolar degeneration in cardiomyocytes, the presence of hepatocyte steatosis, hydropic degeneration and vacuolar degeneration in the liver. Moreover, the nephrotoxicity was analyzed in terms of renal parenchymal edema, renal tubular dilatation with protein casts, glomerular hyalinization, vacuolization of gromeruli, and hyaline droplets degeneration.

The tumor samples were characterized according to the pathological classification. The following parameters were analyzed: (i) the metastasis index, which equaled the total metastatic surface normalized to the total lung surface, (ii) the number of mitotic cells in 5 large fields of view at a magnification of 40X (5HPF-40X), (iii) the apoptosis score estimated by counting the number of apoptotic cells in necrosis-free metastatic foci in the 5HPF-40X images (0—no apoptotic cells, 1—very low score, 2—low score, 3—high score, 4—very high score), (iv) the necrosis index, which equaled the total necrotic surface normalized to the total metastasis surface, and (v) the degenerative changes expressed as a percentage of the cells within the degenerative features in 100 metastatic cells in the 10HPF-40X images.

### Statistics

The statistical significance of the differences between the sphere-treated groups was calculated using a one-way or two-way ANOVA with Bonferroni post hoc correction. The differences between groups were considered significant if the p-value < 0.05.

### Additional methods

A description of the methods used to perform research presented in the additional files is indicated in the Additional file 1: Supplementary methods.

## Supplementary information

**Additional file 1:** Supplementary methods.

**Additional file 2: Table S1.** Analysis of the luminescence intensity of D2F2E2 and D2F2 cells clones transfected with the cDNA encoding luciferase (LUC).

**Additional file 3: Figure S1.** The proliferation of D2F2E2 and D2F2 cells transfected with the cDNA encoding luciferase (LUC) determined by the MTT assay. Cells were seeded at 1 x 10^4^ cells/well and cultured for 72 hours. The graphs present the comparison of the proliferation rate of unmodified cells (control) and (a) D2F2E2/LUC and (b) D2F2/LUC clones selected based on the luminescence intensity. The results are expressed as the mean of three independent experiments ± SEM. (**) indicates statistical significance with p < 0.01 and (***) p < 0.001, ns – not significant. The D2F2E2/LUC #1 and D2F2/LUC #5 clones displaying similar levels of luminescence and proliferation rate were selected for the in vivo studies.

**Additional file 4: Figure S2.** The proliferation of selected clones of D2F2E2/LUC and D2F2/LUC cells determined by MTT assay. Cells were seeded at 1 x 10^4^ cells/well and cultured for 72 hours. The mean absorbance and (± SEM) of at least three independent experiments are shown; (**) indicates statistical significance with p < 0.01.

**Additional file 5: Figure S3.** H&E staining of FFPE sections of organs collected after treatment. Her2(+) D2F2E2 tumor-bearing mice were injected intravenously with a) free Dox, b) PBS and c) Dox-loaded H2.1MS1 spheres according to the schedule presented in Figure 3a. Organs such as the heart, kidney, liver, and lungs were excised on the 20th day, and the samples were stained with H&E. Scale bar: 100 μm.

**Additional file 6: Figure S4.** H&E staining of the heart tissue from tumor-bearing mice after treatment with free Dox at higher magnification. White arrows indicates small vacuoles in cardiomyocytes. Scale bar: 50 μm.

**Additional file 7: Figure S5.** H&E staining of the liver tissue from tumor-bearing mice after treatment with free Dox at higher magnification. Black arrows points out the examples of vacuolar degeneration. Scale bar: 50 μm.

**Additional file 8: Figure S6.** H&E staining of the FFPE sections of kidneys from tumor-bearing mice after treatment with free Dox at higher magnification. Circles indicate renal lesions, such as a) vacuolization of glomeruli (red), b) hyaline droplets degeneration (yellow), c) glomerular hyalinization (black), and d) renal tubular dilatation with protein casts (green). Scale bar: 50 μm.

**Additional file 9: Figure S7.** Iron deposits in spleen collected after treatment. Her2(+) D2F2E2 tumor-bearing mice were injected intravenously with free Dox, PBS, and Dox-loaded H2.1MS1 spheres according to the schedule presented in Figure 4a. Spleens were excised on the 20th day, and the samples were stained with Iron Stain. Deep blue, iron deposits; red, nuclei; pink, background. Scale bar: 100 μm.

**Additional file 10: Figure S8.** The dose-dependent efficacy of Dox delivered in silk spheres in a Her2(+) orthotopic breast cancer model. The D2F2E2 tumors excised 20 days after the beginning of the treatment indicated in Figure 6a.

**Additional file 11: Figure S9.** The dose-dependent therapeutic effect of Dox delivered in silk spheres in a Her2(−) orthotopic breast cancer model. The schematic representation of the treatment course is shown in Figure 6a. a) Kinetics of tumor growth during treatment in a Her2(−) tumor mouse model. b) The D2F2 tumors excised 20 days after the beginning of the treatment. c) Weight of D2F2 tumor-bearing mice during treatment. The data presented are expressed as means ± SEM; ns – not significant.

**Additional file 12: Figure S10.** Histological analysis of lung tissue sections in a model of metastatic breast cancer. Images of H&E-stained samples from mice that developed D2F2E2 tumor metastasis and were utilized in the biodistribution studies. The study was conducted according to the schedule presented in Figure 8a. Scale bar: 200 µm.

**Additional file 13: Table S2.** Raw data of histological analysis of lung tissue sections in a model of breast cancer metastasis. Tumor samples from mice developed Her2(+) D2F2E2 tumor were characterized according to the pathological classification.

**Additional file 14: Figure S11.** Representative images of Ki-67 staining of a) D2F2E2 and b) D2F2 tumors in a model of breast cancer metastasis. Sections of the lungs were labeled using Ki-67 rabbit monoclonal antibody clone SP6, and IHC assay En Vision^TM^ FLEX GV8002. Scale: 20 µm.

**Additional file 15: Figure S12.** Representative images of TUNEL staining of a) D2F2E2 and b) D2F2 tumors in a model of breast cancer metastasis. Sections of the lungs were labeled by the TUNEL technique using the Deadend^TM^ Colorimetric TUNEL System (Promega). The apoptotic cells are indicated by black arrows. Scale: 20 µm.

**Additional file 16: Table S3.** Raw data of histological analysis of lung tissue sections in a model of breast cancer metastasis. Tumor samples from mice developed Her2(−) D2F2 tumor were characterized according to the pathological classification.

**Additional file 17: Figure S13.** The lungs of mice receiving (a) D2F2E2 and (b) D2F2 cells that were excised 20 days after the beginning of the treatment with Dox-loaded H2.1MS1 particles, as indicated in Figure 9. Scale bar: 1 cm.

## Data Availability

The analyzed datasets generated during the current study are available from the corresponding author on reasonable request.

## Abbreviations

DDS: Drug delivery system
Dox: Doxorubicin
PBS: Phosphate buffered saline
LUC: Luciferase
DMEM: Dulbecco’s modified Eagle medium
H&E: Hematoxyline & Eosine
EPR: Enhanced permeability and retention effect
TEM: Tumor microenvironment
