# Supplementary material for: Functionalized silk spheres selectively and effectively deliver a cytotoxic drug to targeted cancer cells in vivo
Source: J Nanobiotechnology. 2020 Dec 1;18:177. doi: 10.1186/s12951-020-00734-y (PMC7709326; doi:10.1186/s12951-020-00734-y)
Supplement: Supplementary file 1 — Additional file 1: Supplementary methods. [file 12951_2020_734_MOESM1_ESM.docx]

Additional Information

**Functionalized Silk Spheres Selectively and Effectively Deliver a Cytotoxic Drug to Targeted Cancer Cells *In Vivo***

*Anna Florczak*, Tomasz Deptuch, Anna Lewandowska, Karolina Penderecka, Elzbieta Kramer, Andrzej Marszalek, Andrzej Mackiewicz, Hanna Dams-Kozlowska**

**ADDITIONAL METHODS**

***Luciferase activity in LUC-modified cells***

The assessment of the luminescence intensity in obtained D2F2E2/LUC and D2F2/LUC clones was assessed using the Luciferase Assay System (Promega, Madison, WI, USA) according to the manufacturer's instructions.

***Cell proliferation study by MTT assay***

For cell proliferation assay, 1 × 10^4^ of D2F2E2/LUC and D2F2/LUC cells per well were seeded onto a 96-well plate and incubated for 72 hours. Next, the MTT test was performed as described previously [[1](#_ENREF_1)]. The absorbance of soluble formazan was proportional to the number of cells, and the absolute absorbance values were compared.

***Histological analysis***

Internal organs, such as the heart, kidneys, lungs, spleen and liver, were excised from the mice, fixed with 10% formalin, and then used for routine histopathological processing. The sections were automatically stained using a standard histological protocol with H&E (Leica Biosystems, Wetzlar, Germany). Samples were visualized under a light microscope (BX53, Olympus Corporation, Tokyo, Japan) and imaged using a microscope mounted digital camera and the program CellSens (Olympus Corporation, Center Valley, PA, USA). To assess treatment-induced systemic toxicity, samples were analyzed in terms of the macroscopic changes in the examined organ sections.

***Iron staining***

Iron staining was performed on FFPE section of spleen using VENTANA BenchMark Special Stain Automated slide stainer (Roche, Basel, Switzerland) and Iron Staining Kit (Roche, Basel, Switzerland). Briefly, Iron Reagent A and Iron Reagent B were added to react with ionic iron in the tissue to produce a bright blue color. Nuclear Fast Red counterstain was applied to provide a contrasting pink to red background.

***Immunohistochemical staining (IHC)***

From lungs, FFPE tissue sections of 4.5 μm were cut, mounted on the adhesive slides and subjected to immunohistochemistry using Ki-67, rabbit monoclonal antibody clone SP6 (Cell Marque, Rocklin, CA, USA) and IHC kit En Vision^TM^ FLEX GV8002 (DAKO, Santa Clara, CA, USA). The result obtained was expressed as a percent of stained cells in five random 40X fields per sample (5HPF - 40X).

***TUNEL assay***

The terminal deoxynucleotidyl transferase dUTP nick end labeling (TUNEL) assay was carried out using Deadend^TM^ Colorimetric TUNEL System (Promega, Madison, WI, USA) according to manufacturer’s instruction. Apoptotic cells were detected after incubation in the 3,3’-diaminobenzidine (DAB) chromogen for approximately 10 min, and then the samples were counterstained with hematoxylin. Apoptotic cells were counted manually on digital images in selected 5HPF - 40X and expressed as the following apoptotic score: 0 – none apoptotic cells, 1 – very low score, 2 – low score, 3 – high score, 4 – very high score.

**ADDITIONAL REFERENCES**

[1] Florczak A, Mackiewicz A, Dams-Kozlowska H. Functionalized spider silk spheres as drug carriers for targeted cancer therapy. Biomacromolecules. 2014;15:2971-81.
