# Supplementary material for: Functionalized silk spheres selectively and effectively deliver a cytotoxic drug to targeted cancer cells in vivo
Source: J Nanobiotechnology. 2020 Dec 1;18:177. doi: 10.1186/s12951-020-00734-y (PMC7709326; doi:10.1186/s12951-020-00734-y)
Supplement: Supplementary file 2 — Additional file 2: Table S1. Analysis of the luminescence intensity of D2F2E2 and D2F2 cells clones transfected with the cDNA encoding luciferase (LUC). [file 12951_2020_734_MOESM2_ESM.docx]

| Cell line | # Clone | Luminescence intensity [U] |
| --- | --- | --- |
| D2F2E2/LUC | 1 | 11,538,765 |
|  | 2 | 2,564,732 |
|  | 4 | 3,977,524 |
|  | 8 | 15,723,310 |
| D2F2/LUC | 2 | 69,269,000 |
|  | 5 | 6,899,177 |
|  | 15 | 4,295,294 |
|  | 16 | 6,476,427 |

**Additional Table S1. Analysis of the luminescence intensity of D2F2E2 and D2F2 cells clones transfected with the cDNA encoding luciferase (LUC).**
