# Supplementary figures and images for: Functionalized silk spheres selectively and effectively deliver a cytotoxic drug to targeted cancer cells in vivo
Source: J Nanobiotechnology. 2020 Dec 1;18:177. doi: 10.1186/s12951-020-00734-y (PMC7709326; doi:10.1186/s12951-020-00734-y)

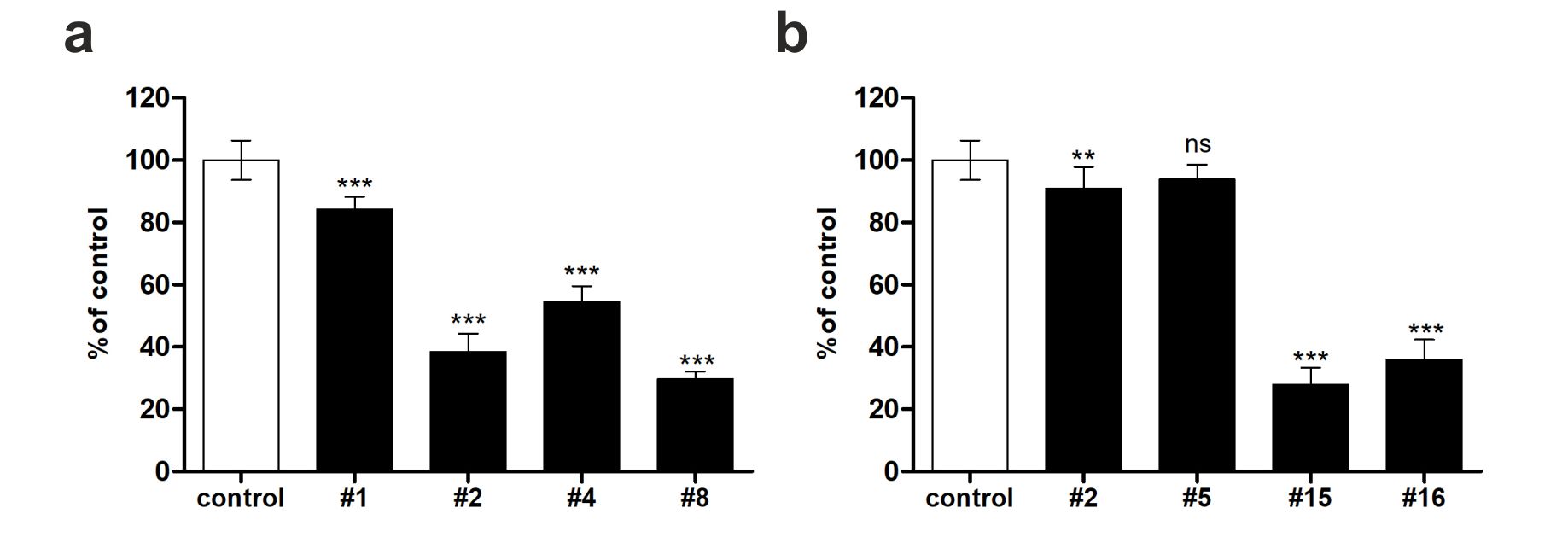

Supplement: Supplementary file 3 — Additional file 3: Figure S1. The proliferation of D2F2E2 and D2F2 cells transfected with the cDNA encoding luciferase (LUC) determined by the MTT assay. Cells were seeded at 1 x 10^4^ cells/well and cultured for 72 hours. The graphs present the comparison of the proliferation rate of unmodified cells (control) and (a) D2F2E2/LUC and (b) D2F2/LUC clones selected based on the luminescence intensity. The results are expressed as the mean of three independent experiments ± SEM. (**) indicates statistical significance with p < 0.01 and (***) p < 0.001, ns – not significant. The D2F2E2/LUC #1 and D2F2/LUC #5 clones displaying similar levels of luminescence and proliferation rate were selected for the in vivo studies. [file 12951_2020_734_MOESM3_ESM.jpg]

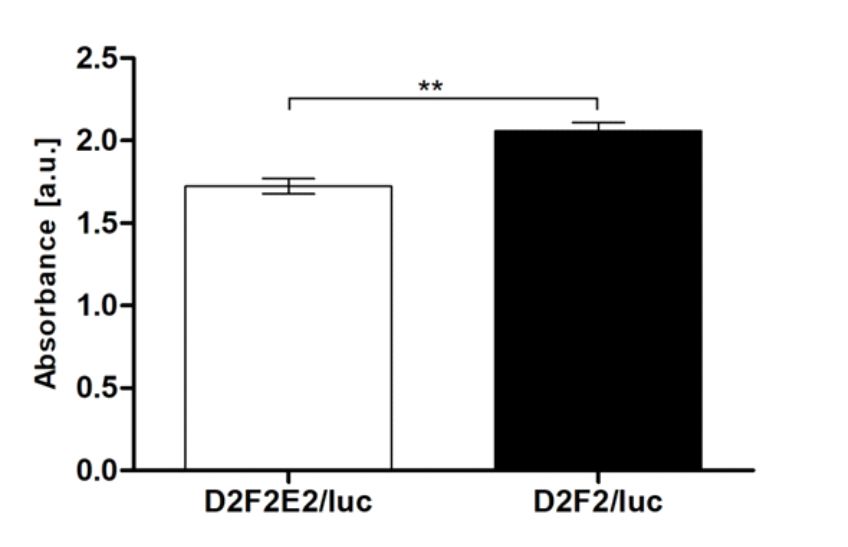

Supplement: Supplementary file 4 — Additional file 4: Figure S2. The proliferation of selected clones of D2F2E2/LUC and D2F2/LUC cells determined by MTT assay. Cells were seeded at 1 x 10^4^ cells/well and cultured for 72 hours. The mean absorbance and (± SEM) of at least three independent experiments are shown; (**) indicates statistical significance with p < 0.01. [file 12951_2020_734_MOESM4_ESM.jpg]

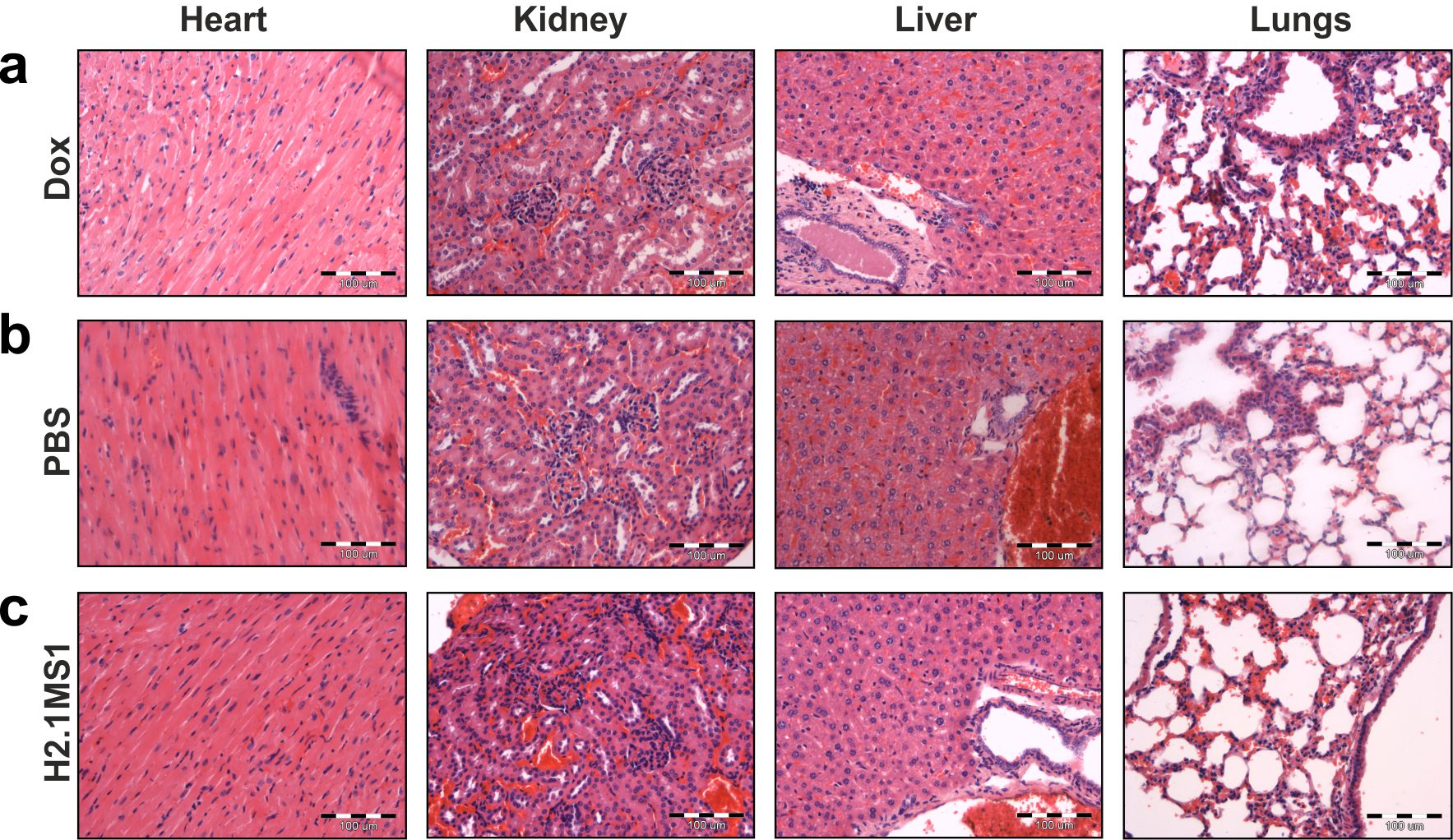

Supplement: Supplementary file 5 — Additional file 5: Figure S3. H&E staining of FFPE sections of organs collected after treatment. Her2(+) D2F2E2 tumor-bearing mice were injected intravenously with a) free Dox, b) PBS and c) Dox-loaded H2.1MS1 spheres according to the schedule presented in Figure 3a. Organs such as the heart, kidney, liver, and lungs were excised on the 20th day, and the samples were stained with H&E. Scale bar: 100 μm. [file 12951_2020_734_MOESM5_ESM.jpg]

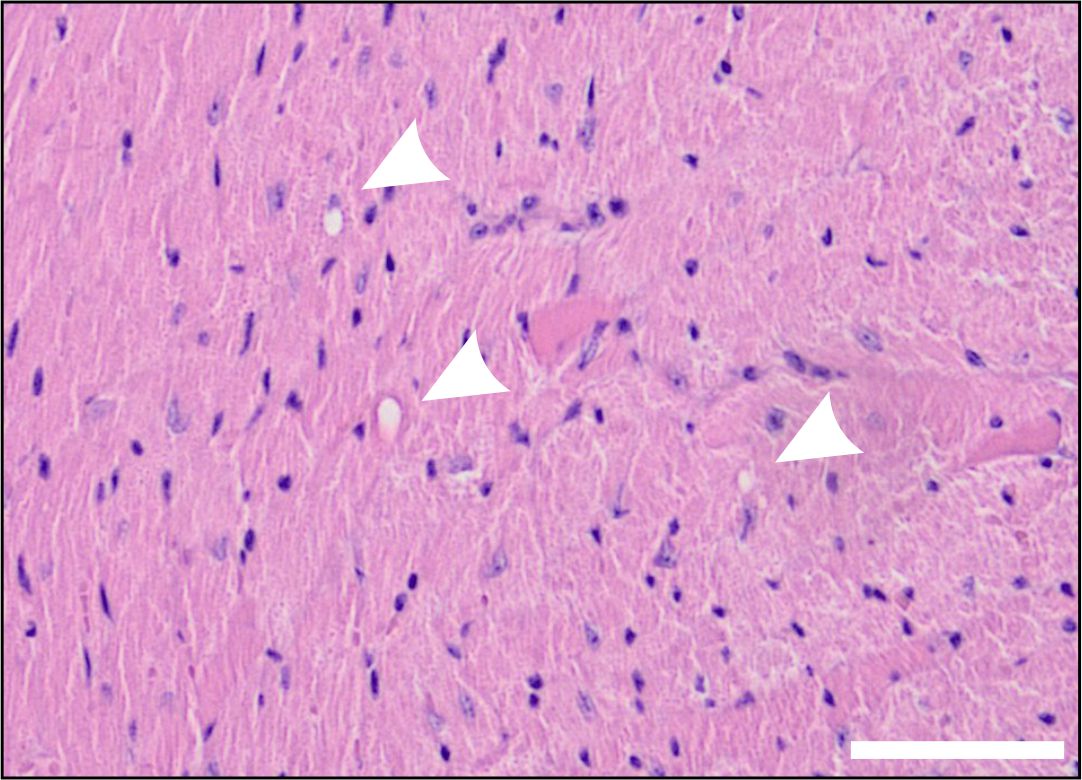

Supplement: Supplementary file 6 — Additional file 6: Figure S4. H&E staining of the heart tissue from tumor-bearing mice after treatment with free Dox at higher magnification. White arrows indicates small vacuoles in cardiomyocytes. Scale bar: 50 μm. [file 12951_2020_734_MOESM6_ESM.jpg]

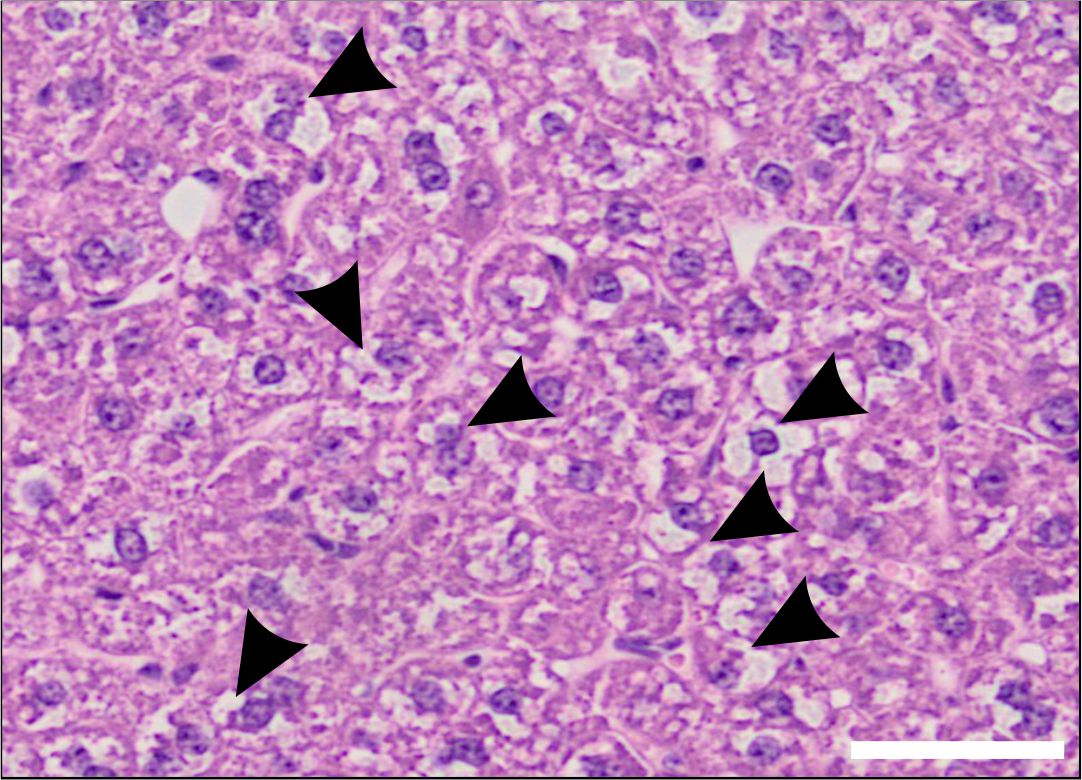

Supplement: Supplementary file 7 — Additional file 7: Figure S5. H&E staining of the liver tissue from tumor-bearing mice after treatment with free Dox at higher magnification. Black arrows points out the examples of vacuolar degeneration. Scale bar: 50 μm. [file 12951_2020_734_MOESM7_ESM.jpg]

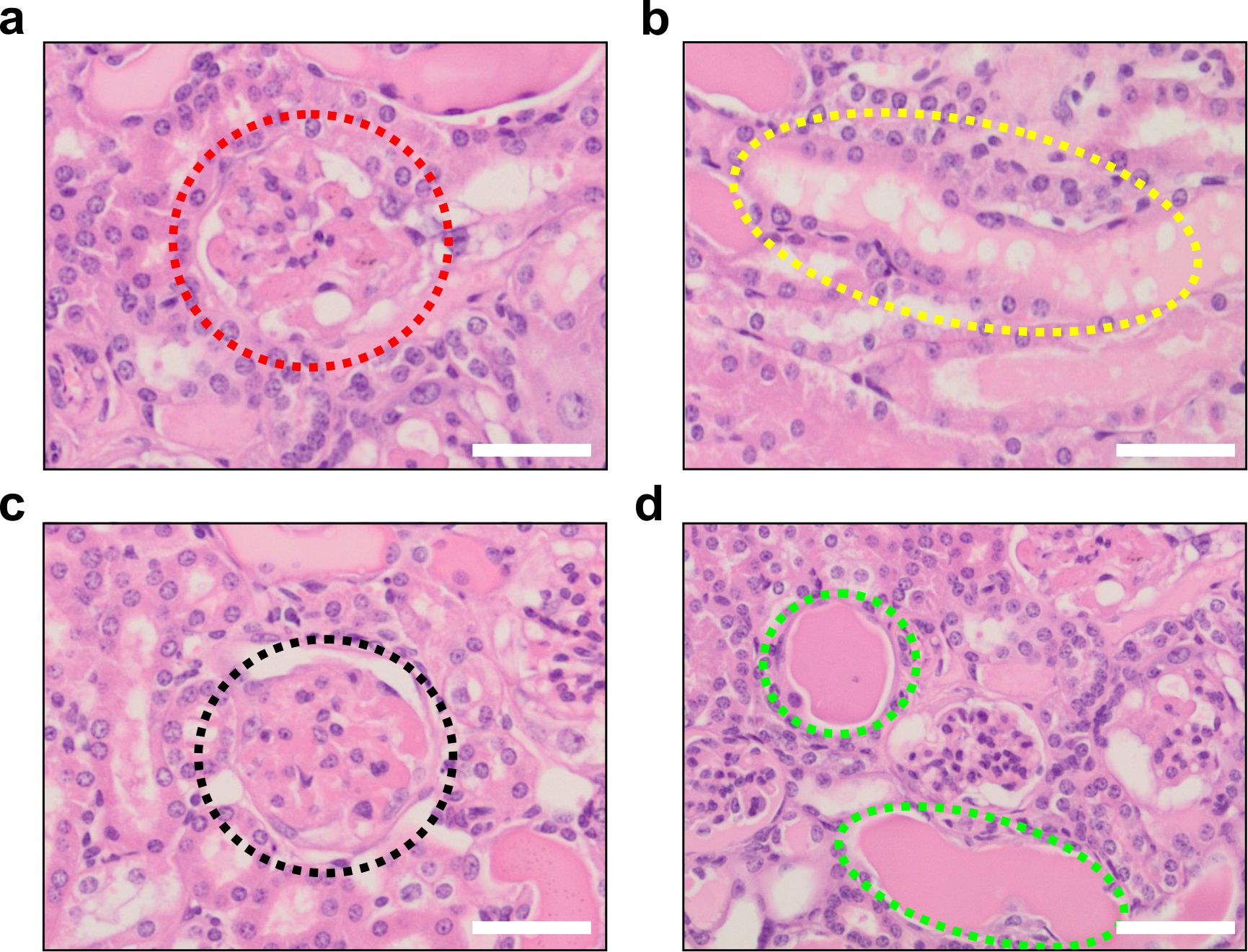

Supplement: Supplementary file 8 — Additional file 8: Figure S6. H&E staining of the FFPE sections of kidneys from tumor-bearing mice after treatment with free Dox at higher magnification. Circles indicate renal lesions, such as a) vacuolization of glomeruli (red), b) hyaline droplets degeneration (yellow), c) glomerular hyalinization (black), and d) renal tubular dilatation with protein casts (green). Scale bar: 50 μm. [file 12951_2020_734_MOESM8_ESM.jpg]

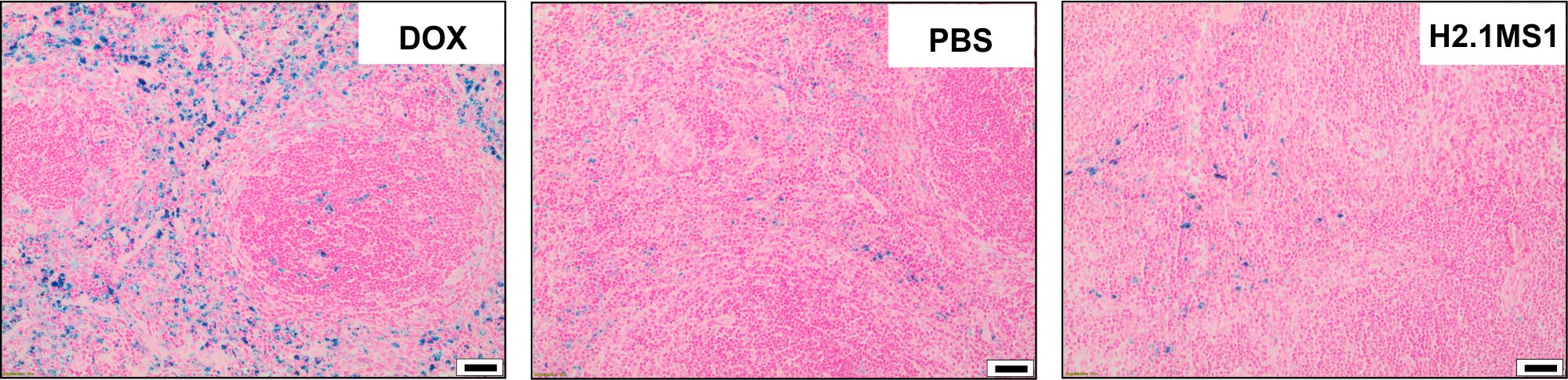

Supplement: Supplementary file 9 — Additional file 9: Figure S7. Iron deposits in spleen collected after treatment. Her2(+) D2F2E2 tumor-bearing mice were injected intravenously with free Dox, PBS, and Dox-loaded H2.1MS1 spheres according to the schedule presented in Figure 4a. Spleens were excised on the 20th day, and the samples were stained with Iron Stain. Deep blue, iron deposits; red, nuclei; pink, background. Scale bar: 100 μm. [file 12951_2020_734_MOESM9_ESM.jpg]

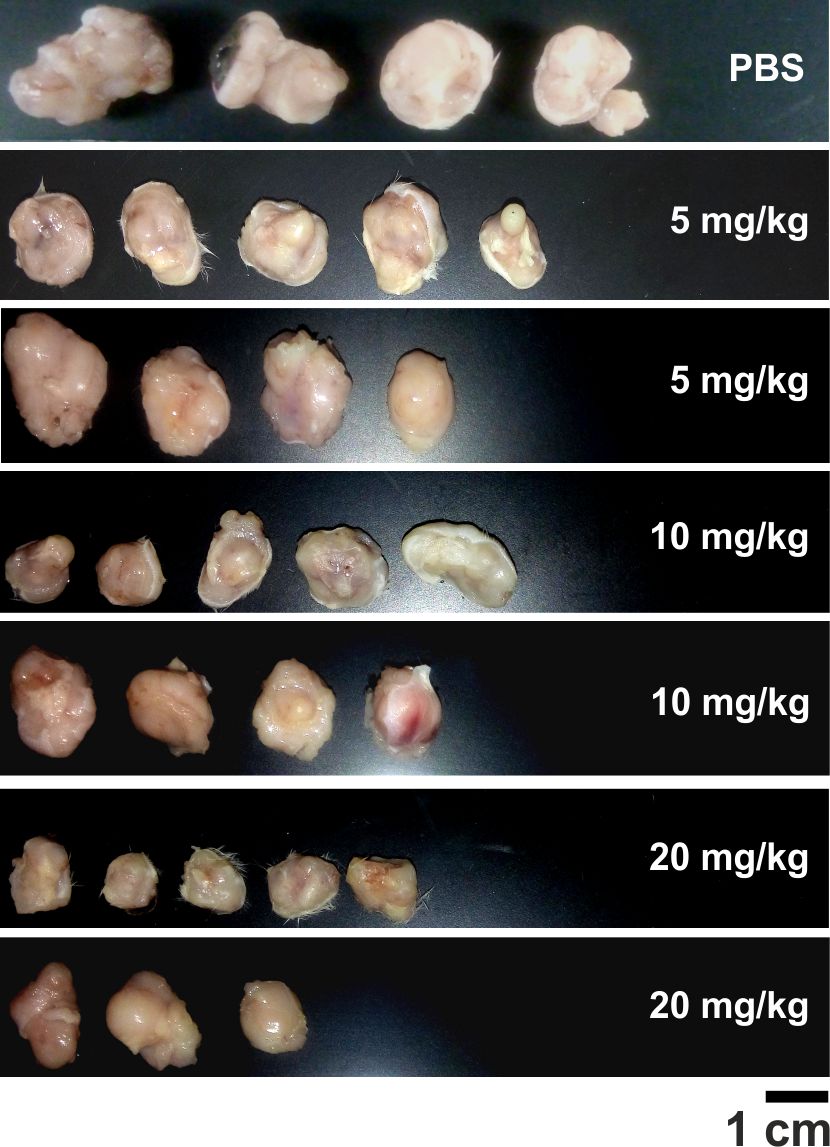

Supplement: Supplementary file 10 — Additional file 10: Figure S8. The dose-dependent efficacy of Dox delivered in silk spheres in a Her2(+) orthotopic breast cancer model. The D2F2E2 tumors excised 20 days after the beginning of the treatment indicated in Figure 6a. [file 12951_2020_734_MOESM10_ESM.jpg]

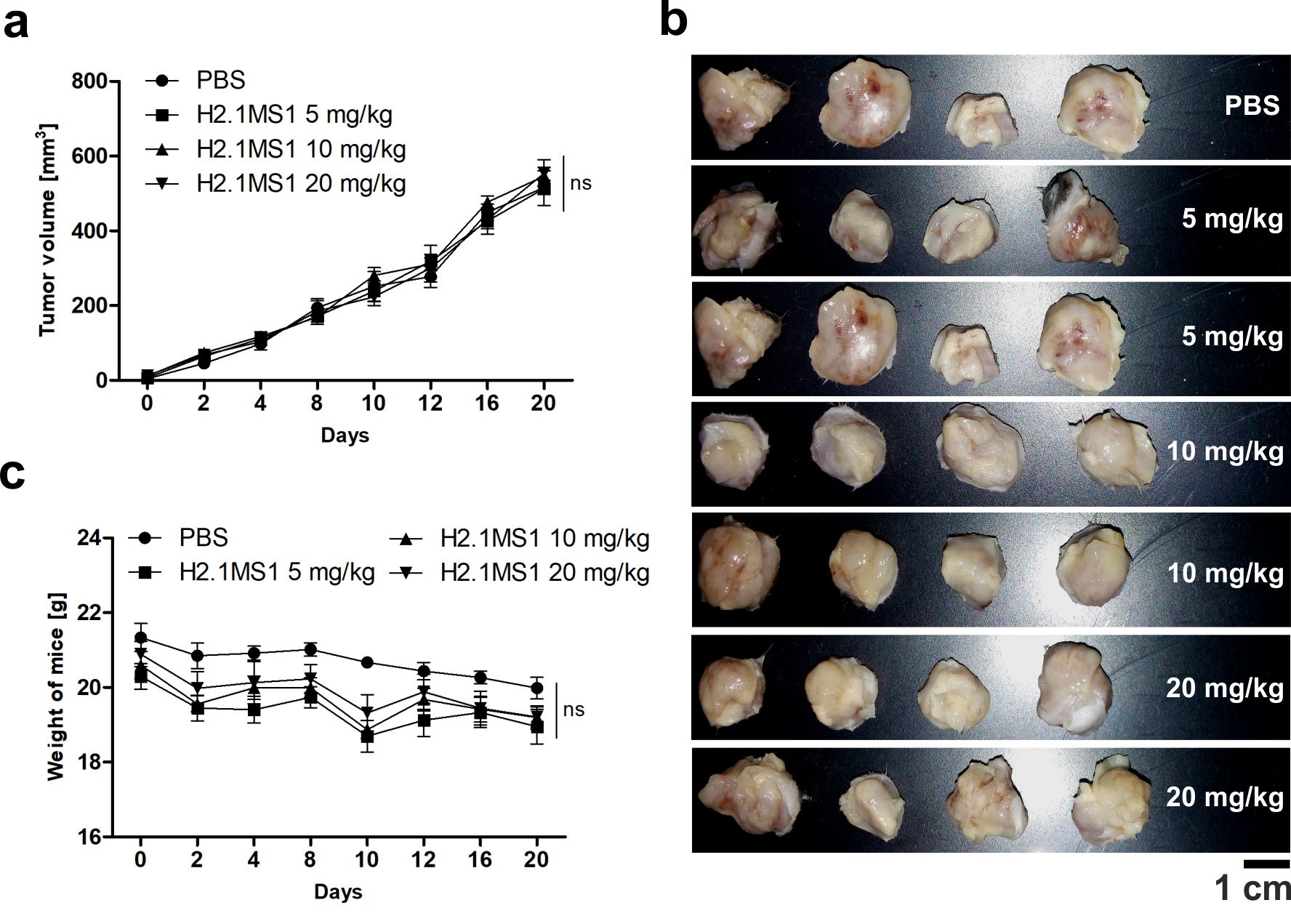

Supplement: Supplementary file 11 — Additional file 11: Figure S9. The dose-dependent therapeutic effect of Dox delivered in silk spheres in a Her2(−) orthotopic breast cancer model. The schematic representation of the treatment course is shown in Figure 6a. a) Kinetics of tumor growth during treatment in a Her2(−) tumor mouse model. b) The D2F2 tumors excised 20 days after the beginning of the treatment. c) Weight of D2F2 tumor-bearing mice during treatment. The data presented are expressed as means ± SEM; ns – not significant. [file 12951_2020_734_MOESM11_ESM.jpg]

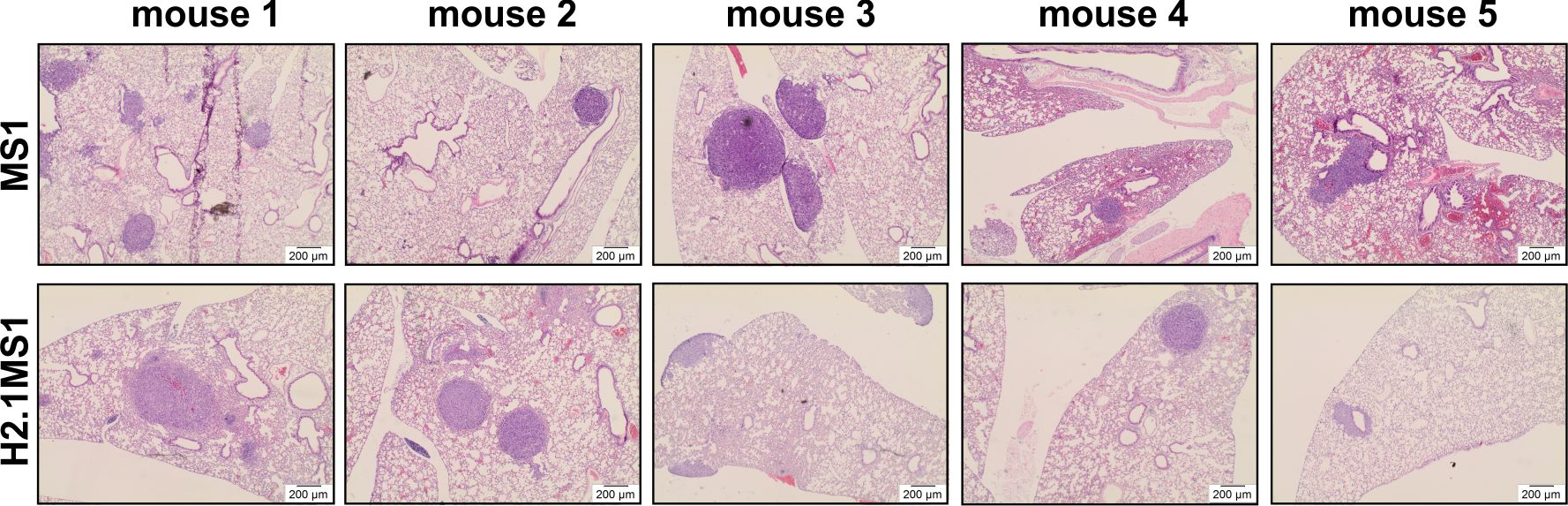

Supplement: Supplementary file 12 — Additional file 12: Figure S10. Histological analysis of lung tissue sections in a model of metastatic breast cancer. Images of H&E-stained samples from mice that developed D2F2E2 tumor metastasis and were utilized in the biodistribution studies. The study was conducted according to the schedule presented in Figure 8a. Scale bar: 200 µm. [file 12951_2020_734_MOESM12_ESM.jpg]

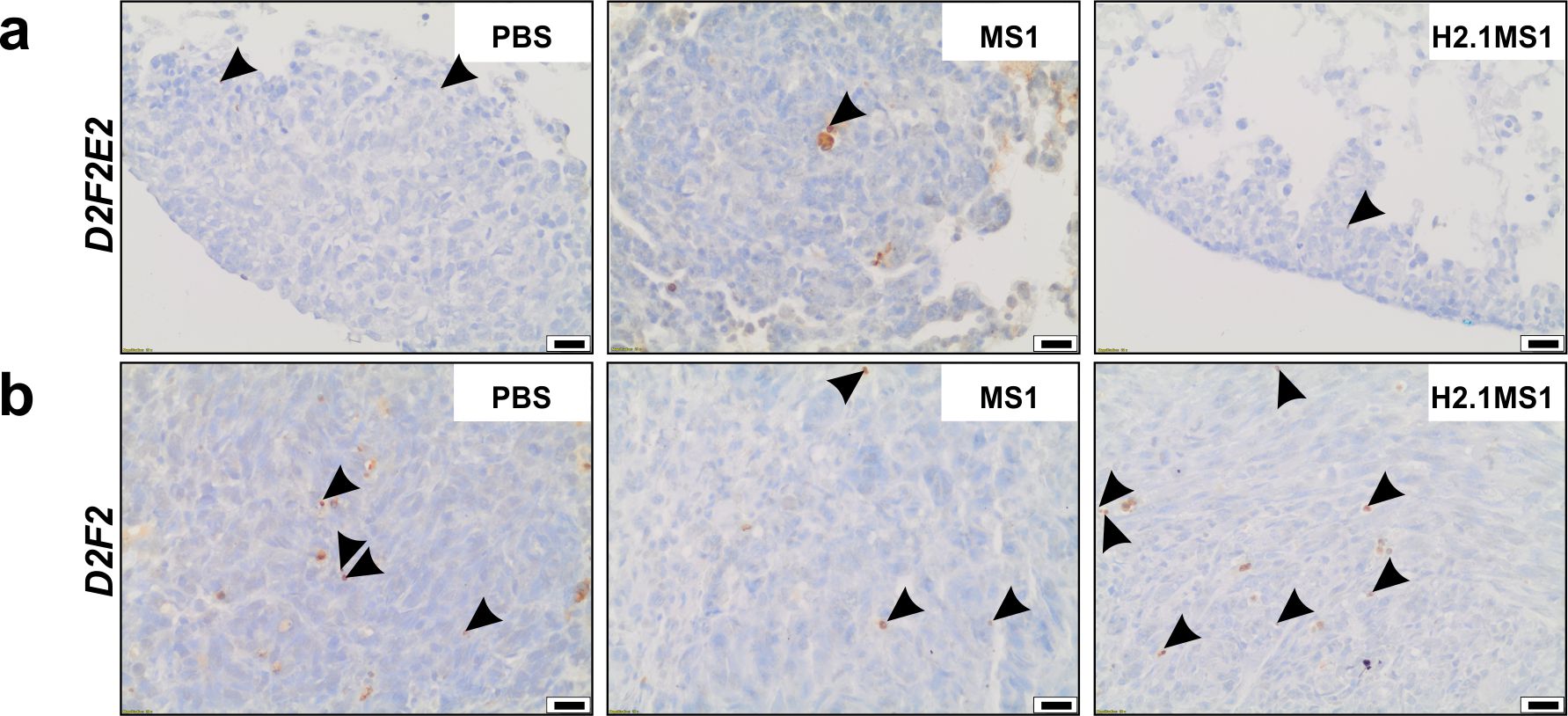

Supplement: Supplementary file 14 — Additional file 14: Figure S11. Representative images of Ki-67 staining of a) D2F2E2 and b) D2F2 tumors in a model of breast cancer metastasis. Sections of the lungs were labeled using Ki-67 rabbit monoclonal antibody clone SP6, and IHC assay En VisionTM FLEX GV8002. Scale: 20 µm. [file 12951_2020_734_MOESM14_ESM.jpg]

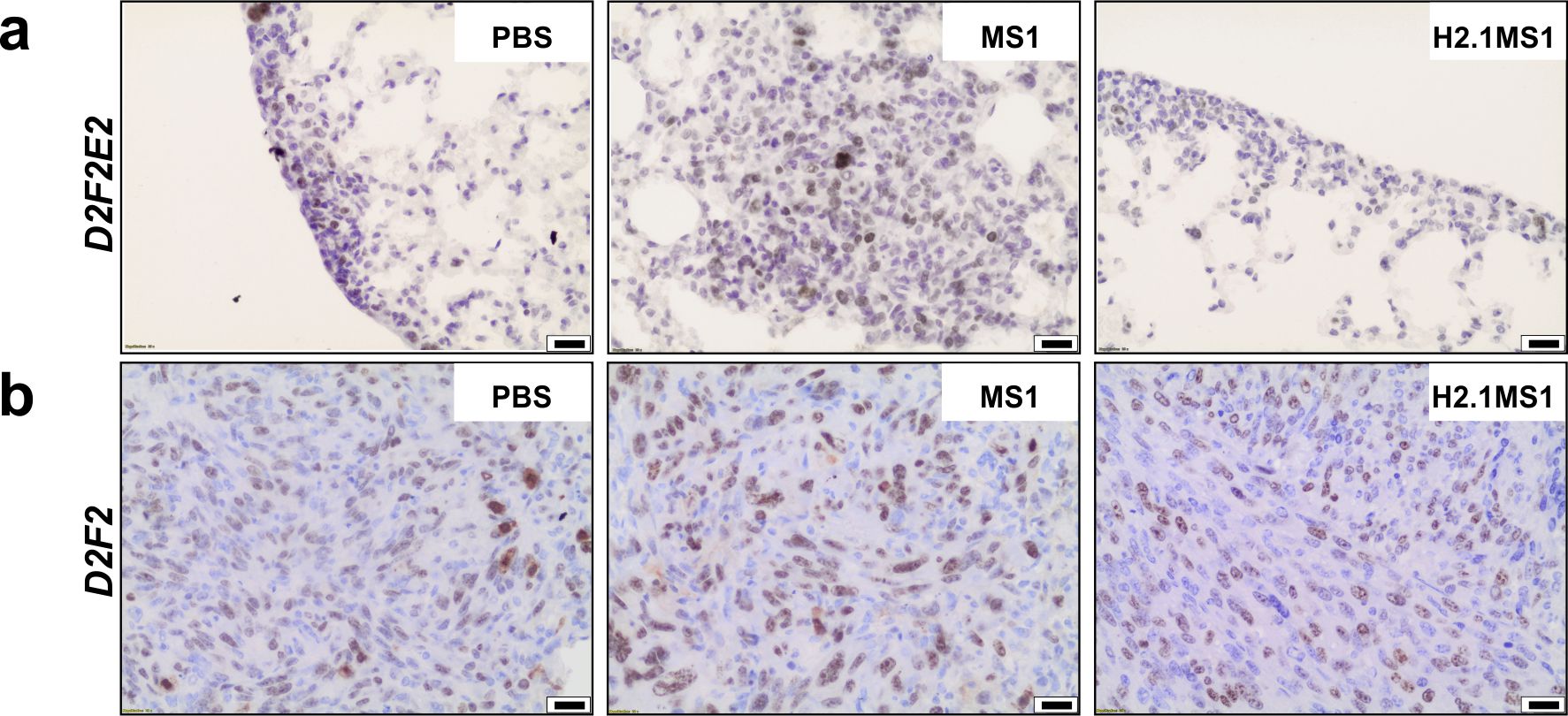

Supplement: Supplementary file 15 — Additional file 15: Figure S12. Representative images of TUNEL staining of a) D2F2E2 and b) D2F2 tumors in a model of breast cancer metastasis. Sections of the lungs were labeled by the TUNEL technique using the DeadendTM Colorimetric TUNEL System (Promega). The apoptotic cells are indicated by black arrows. Scale: 20 µm. [file 12951_2020_734_MOESM15_ESM.jpg]

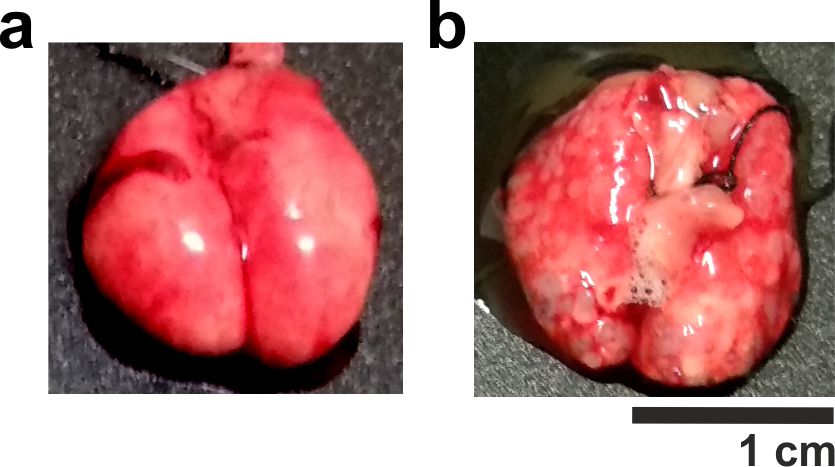

Supplement: Supplementary file 17 — Additional file 17: Figure S13. The lungs of mice receiving (a) D2F2E2 and (b) D2F2 cells that were excised 20 days after the beginning of the treatment with Dox-loaded H2.1MS1 particles, as indicated in Figure 9. Scale bar: 1 cm. [file 12951_2020_734_MOESM17_ESM.jpg]
