# Supplementary material for: Functionalized silk spheres selectively and effectively deliver a cytotoxic drug to targeted cancer cells in vivo
Source: J Nanobiotechnology. 2020 Dec 1;18:177. doi: 10.1186/s12951-020-00734-y (PMC7709326; doi:10.1186/s12951-020-00734-y)
Supplement: Supplementary file 13 — Additional file 13: Table S2. Raw data of histological analysis of lung tissue sections in a model of breast cancer metastasis. Tumor samples from mice developed Her2(+) D2F2E2 tumor were characterized according to the pathological classification. [file 12951_2020_734_MOESM13_ESM.docx]

**Additional Table S2. Raw data of histological analysis of lung tissue sections in a model of breast cancer metastasis.** Tumor samples from mice developed Her2(+) D2F2E2 tumor were characterized according to the pathological classification as indicated in Materials and Methods section.

|  | Total lung surface | Metastasis surface | Metastasis index | Number of mitoses  (5HPF-40X) | | Apoptosis (Score) | | Necrosis surface | Necrosis index | % of degenerative cells |
| --- | --- | --- | --- | --- | --- | --- | --- | --- | --- | --- |
|  |  |  |  | HE | IHC  (Ki57) | HE | TUNEL |  |  |  |
| PBS.1 | 755,444 | 12,958 | 1.7 | 7,5,5,1,3  =21 | - | 1 | 1 | 0 | 0 | 0, 0, 0, 0, 0, 0, 0, 0, 0,0  0% |
| PBS.2 | 1,340,707 | 4,835 | 0.36 | 1,1,7,10,9  =28 | 5% (1 HPF) | 1 | 1 | 0 | 0 | 0, 1, 1, 0, 0, 0, 0, 0, 0, 1 = 0,3% |
| PBS.3 | 1,077,396 | 1,453 | 0.13 | 3, 8, 10, 2, 5  =28 | 15% (1 HPF) | 1 | 0 | 0 | 0 | 1, 2, 0, 2, 5, 0, 0, 1, 1, 1 = 1,3% |
| MS1.1 | 2,001,170 | 47,818 | 2.4 | 1,4,4,1,2  =12 | - | 1 | 1 | 0 | 0 | 0,1,0,1,1,1, 1, 0, 1, 0  = 0,6% |
| MS1.2 | 1,451,670 | 12,070 | 0.83 | 5, 3, 2, 2, 0  =12 | 6% (5 HPF) | 1 | 1 | 0 | 0 | 1, 1, 2, 5, 40, 1, 20, 0, 1, 5 = 7,6% |
| MS1.3 | 1,423,500 | 5,187 | 0.36 | 0, 1, 3, 2, 2  =8 | 16% (5 HPF) | 1 | 2 | 0 | 0 | 0, 1, 1, 2, 30, 0, 0, 0,10,10 = 5,4% |
| H2.1MS1.1 | 1,622,959 | 3,780 | 0.2 | 2,2,4,3,1  =12 | - | 1 | No metastatic foci | 0 | 0 | less than 100 cells in 1HPF or No metastatic foci |
| H2.1MS1.2 | 1,284,925 | 57 | 0.004 | 1 (1HPF) | No metastatic foci | 1 | 1 | 0 | 0 | 6% (1HPF) or less than 100 cells in 1HPF or No metastatic foci |
| H2.1MS1.3 | 2,389,764 | 670 | 0.03 | 1 (1HPF) | 5%  (1 HPF) | 1 | No metastatic foci | 0 | 0 | less than 100 cells in 1HPF or No metastatic foci |
