# Supplementary material for: Functionalized silk spheres selectively and effectively deliver a cytotoxic drug to targeted cancer cells in vivo
Source: J Nanobiotechnology. 2020 Dec 1;18:177. doi: 10.1186/s12951-020-00734-y (PMC7709326; doi:10.1186/s12951-020-00734-y)
Supplement: Supplementary file 16 — Additional file 16: Table S3. Raw data of histological analysis of lung tissue sections in a model of breast cancer metastasis. Tumor samples from mice developed Her2(−) D2F2 tumor were characterized according to the pathological classification. [file 12951_2020_734_MOESM16_ESM.docx]

**Additional Table S3. Raw data of histological analysis of lung tissue sections in a model of breast cancer metastasis.** Tumor samples from mice developed Her2(-) D2F2 tumor were characterized according to the pathological classification as indicated in Materials and Methods section.

|  | Total lung surface | Metastasis surface | Metastasis index | Number of mitoses  (5HPF-40X) | | Apoptosis (Score) | | Necrosis surface | Necrosis index | % of degenerative cells |
| --- | --- | --- | --- | --- | --- | --- | --- | --- | --- | --- |
|  |  |  |  | HE | IHC  (Ki57) | HE | TUNEL |  |  |  |
| PBS.1 | 1,261,702 | 416,671 | 33.02 | 13, 16, 8, 10, 9 = 56 | 34% (5 HPF) | 2 | 2 | 35,374 | 8.49 | 1, 0, 1, 0, 0, 0, 0, 1, 1, 1  = 0,5% |
| PBS.2 | 1,502,929 | 728,103 | 48.45 | 5, 11, 13, 13, 10 = 52 | 40% (5 HPF) | 2 | 2 | 83,738 | 11.5 | 0, 0, 0, 0, 0, 0, 0, 0, 0,0  0% |
| PBS.3 | 1,355,387 | 142,217 | 10.49 | 9, 3, 4, 9, 6  = 31 | 35% (5 HPF) | 2 | 2 | 9,892 | 6.96 | 0, 0, 0, 0, 0, 0, 0, 0, 0,0  0% |
| MS1.1 | 1,643,628 | 50,360 | 3.06 | 3, 3, 9, 2, 7  = 24 | 48% (5 HPF) | 1 | 1 | 868 | 1.72 | 10, 1, 0, 3, 1, 2, 10, 80, 1, 10 = 11,8% |
| MS1.2 | 1,944,029 | 462,934 | 23.81 | 11, 11, 15, 9, 10 = 56 | 42% (5 HPF) | 2 | 2 | 3,745 | 0.81 | 1, 1, 2, 1, 2, 1, 10, 0, 1, 0  = 1,9% |
| MS1.3 | 1,601,645 | 485,614 | 30.32 | 4, 2, 7, 2, 8  = 23 | 37% (5 HPF) | 2 | 2 | 3,501 | 0.72 | 1, 2, 20, 10, 2, 2, 2, 1, 1, 0 = 4,1% |
| H2.1MS1.1 | 2,406,251 | 208,698 | 8.67 | 8, 3, 7, 8, 4  = 30 | 20% (5 HPF) | 3 | 2 | 4,355 | 2.09 | 0, 10, 1, 30, 1, 30, 15, 1, 0, 5 = 9,3% |
| H2.1MS1.2 | 2,781,452 | 878,635 | 31.59 | 5, 9, 6, 9, 5  = 34 | 22% (5 HPF) | 2 | 2 | 89,637 | 10.2 | 1, 1, 0, 0, 5, 2, 1, 15, 5, 10 = 4% |
| H2.1MS1.3 | 3,302,270 | 1,827,529 | 55.34 | 4, 3, 3 , 1, 2  = 13 | 17% (5 HPF) | 2 | 2 | 60,347 | 3.3 | 0, 0, 0, 2, 1, 2, 1, 0, 1, 2  = 0,9% |
